# Cell transmission model of dynamic assignment for urban rail transit networks

**DOI:** 10.1371/journal.pone.0188874

**Published:** 2017-11-30

**Authors:** Guangming Xu, Shuo Zhao, Feng Shi, Feilian Zhang

**Affiliations:** 1 School of Civil Engineering, Central South University, Changsha, Hunan, China; 2 School of Traffic and Transportation Engineering, Central South University, Changsha, Hunan, China; Beihang University, CHINA

## Abstract

For urban rail transit network, the space-time flow distribution can play an important role in evaluating and optimizing the space-time resource allocation. For obtaining the space-time flow distribution without the restriction of schedules, a dynamic assignment problem is proposed based on the concept of continuous transmission. To solve the dynamic assignment problem, the cell transmission model is built for urban rail transit networks. The priority principle, queuing process, capacity constraints and congestion effects are considered in the cell transmission mechanism. Then an efficient method is designed to solve the shortest path for an urban rail network, which decreases the computing cost for solving the cell transmission model. The instantaneous dynamic user optimal state can be reached with the method of successive average. Many evaluation indexes of passenger flow can be generated, to provide effective support for the optimization of train schedules and the capacity evaluation for urban rail transit network. Finally, the model and its potential application are demonstrated via two numerical experiments using a small-scale network and the Beijing Metro network.

## Introduction

The development of urban rail transit systems (URTS) has been proposed as a coping strategy to relieve traffic congestion around the world. A URTS not only has the properties of large capacity with less land occupation, but also has the advantages of energy conservation, environmental protection, high safety and reliability. It is a sustainable transportation mode.

Urban traffic demand is known as the time-varying origin–destination (O-D) demand. For example, during the morning rush hour, passengers gather from places of residence to their work place; during the afternoon rush hour, passengers disperse from their work place to other places. The time-varying demand makes the URTS operation complex.

The core of the URTS operation is the train schedule. A high-quality schedule must fit the time-varying flow, i.e., it should have a high train frequency when the flows are large in some time intervals. Train schedule design of urban rail network is a rather complex problem with considering the time-varying demand. To simplify the schedule design problem, it is always divided into the schedule design for each single rail line [1–2]. Niu and Zhou [1] used the time-varying O-D demand for a single line and optimized its schedule. Shi *et al*. [2] optimized the frequency setting, timetabling and the rolling stock circulation for a single line based on the time-varying section flow. As the time-varying O-D demands or section flows for a single line include the passengers taking this rail line directly and transferring from other lines, and it is need to design a method for obtaining the reasonable time-varying O-D demands or section flows for each single line, it motivates us to study the dynamic assignment for urban rail transit network (DAURTN), which can approximately estimate the space-time flow distribution in the urban rail network and be used to evaluate and optimize the space-time resource allocation for a URTS.

The space-time flow distribution in the urban rail network can be obtained by the traditional method of investigation and statistics [3–4], on condition that the rail networks and time-varying O-D demands are stable, but the workload is very heavy and the results are restricted by the schedule and transportation capacity distribution. Another method for solving the problem of DAURTN is the schedule-based transit assignment models [5–14]. When considering the travel behaviors such as capacity constraints, space priority, first-come-first-serve (FCFS) and congestion effects together, the scales of networks studied in the existing literature are very small, and it is difficult to solve the realistic scale networks in the schedule-based transit assignment. Moreover, it is obvious that the results are also restricted by the schedule, which make the time-varying section flow be discrete.

To fill up the research gap, we proposed the concept of continuous transmission, which means that the urban rail line can serve passengers at any time and it is not restricted by the train’s schedule times. The concept of continuous transmission is similar to the continuous network in continuum equilibrium traffic assignment models [15–18]. The difference between the two concepts is that, for the concept of continuous transmission, the line service is continuous in the time axis and there is no schedule, while the concept of continuous network means the road network is approximated as a continuum [15]. With the concept of continuous transmission and maximal rail line capacity constraints, the problem of DAURTN is a new challenging problem considering the travel behaviors such as the capacity constraints, space priority, FCFS and congestion effects together. This problem of DAURTN plays an important role in evaluating and optimizing the schedule for a URTS, as well as optimizing the frequency and the rolling stock circulation, and evaluating the transportation capacity for urban rail networks. For solving the above problem, we design a cell transmission model (CTM).

The dynamic assignment problem (DAP) derives from urban road traffic assignment research, which can reflect how vehicles make appropriate route choices at any time according to the current road flow state, and also can depict travel choice behaviors more precisely. For solving the DAP, many works [19–30] in the literature have studied how to establish and solve models, and there are two classes of models, i.e., instantaneous dynamic route choice model and idea dynamic route choice model [22]. To solve the DAP considering the capacity constraints, there are also abundant studies [31–37].

The CTM is a practical method for dynamic traffic assignment, and has a relatively high solving efficiency. Danganzo [38–39] started to use the concept of a cellular automaton, and proposed a CTM to study the dynamic traffic problem. Lo and Szeto [40] developed a cell-based dynamic traffic assignment formulation which follows the ideal dynamic user optimal principle. This formulation automatically satisfies the first-in-first-out (FIFO) conditions as a result of encapsulating CTM, and can capture dynamic traffic phenomena such as shockwaves, queue formation and dissipation. Szeto and Lo [41] further considered departure time choice and elastic demand. For dynamic traffic assignment, they generally assume that traffic flow behaviors follow the FIFO principle, so they apply the CTM to solve the problem. To follow FIFO, they divide the flow into small groups according to different routes, and make them outflow from current cells on the basis of their proportion of the gross, but neglect the time sequence when they flow into the cells. FIFO is also discussed in Daganzo [39], Lo and Szeto [40], Carey [42–43], Blumberg and Bar-Gera [44], Long *et al*. [45] and Carey *et al*. [46]. In particular, Carey *et al*. [46] indicated that the usual recommended method for preserving FIFO will ensure FIFO for each cell taken separately, but does not fully ensure FIFO in the transition between cells or for links or for routes, and the paper is concerned with how to implement FIFO in the CTM. However, the CTM is not used to solve the transit assignment problem.

For transit assignment problem, there are two categories: one is frequency-based, and the other is schedule-based. The latter considers time-varying O-D demand. Tong and Wong [5] proposed a schedule-based, stochastic, dynamic transit assignment model, and a stochastic minimum path is generated by a specially developed branch and bound algorithm. Nuzzolo *et al*. [6] developed a dynamic process assignment model, both within-day and day-to-day, and tested it on a realistically sized network to verify its applicability for operations planning. Nguyen *et al*. [7] presented a new graph theoretic framework for the passenger assignment problem that encompassed the departure time and the route choice. The implicit FIFO access to transit lines was taken into account by the concept of available capacity. Poon *et al*. [8] proposed a predictive transit dynamic user equilibrium model, and the generalised cost function encompassed four components: in-vehicle time, waiting time, walking time, and a line change penalty. Passengers queued at platforms under the single channel first-in-first-out discipline. By using time-increment simulation, the passenger demand was loaded onto the network and the available capacity of each vehicle was updated dynamically. Hamdouch and Lawphongpanich [9] and Hamdouch *et al*. [10–11] proposed a user equilibrium transit assignment model that took into account transit schedules and individual vehicle capacities explicitly. When loading a vehicle, on-board passengers continuing to the next stop had priority and waiting passengers could be loaded on a FCFS or in a random manner. Sumalee *et al*. [12] proposed a stochastic dynamic transit assignment model with an explicit seat allocation process. Two priority rules were assumed in the seat allocation simulation: passengers arriving earlier at a stop can access the available seats prior to those arriving later; standing passengers already on-board can access the available seats prior to those just boarding at the stop/station. Zhang *et al*. [13] proposed a new multi-class user reliability-based dynamic transit assignment model, and the in-vehicle capacity constraint for random passenger demand was handled by an in-vehicle congestion parameter. Nuzzolo *et al*. [14] presented a schedule-based dynamic assignment model for transit networks, which took into account congestion through explicit vehicle capacity constraints, and solved the queue formation and dispersion through FCFS rules, the failure-to-board experience, as well as experienced LoS attributes.

In order to obtain the space-time flow distribution in the urban rail network, without the restriction of schedules, we proposed the concept of continuous transmission for DAURTN. To solve the problem of DAURTN based on the continuous transmission and maximal line capacity constraints, we design the cell transmission mechanism, and develop the CTM. In the construction of the cell transmission rules, the model considers the priority principle, the queuing process, the capacity constraint, and congestion effect. We design an efficient algorithm for solving the shortest path in the urban rail network, which decreases the computation cost of the algorithm for the CTM to be implemented on a large-scale network. Using the method of successive average (MSA), the instantaneous dynamic user optimal state can be reached. Many important indexes are generated by the CTM, which provides the effective support for the optimization of train schedules and the capacity evaluation for the urban rail transit network. We take a small-scale network and the Beijing Metro network as two numerical examples to show the model and its potential application.

## Nomenclatures

*n*: total number of stations;
*m*: total number of lines;
*s*^*u*^: the *u*th station;
*S*: set of stations;
*l*: the *l*th line;
*U*: the up line direction;
*D*: the down line direction;
*d*: the direction variable (*d* ∈ {U,D});
Ω: set of directional lines;
*L*_*ld*_: direction *d* of line *l* in Ω;
$s_{ld}^{i}$: the *i*th platform in the direction *d* of line *l*;
*n*(*l*): number of stations of line *l*;
$\left(s_{ld}^{i},s_{ld}^{i+1}\right)$: the *i*th line section in the direction *d* of line *l* (*i* ≤ *n*(*l*) − 1);
*E*: set of sections;
${\bar{s}}_{ld}^{i}$: corresponding station of platform $s_{ld}^{i}$;
*S*(*s*^*u*^): set of corresponding platforms of station *s*^*u*^;
$d(s_{ld}^{i},s_{ld}^{i+1})$: mileage of the section $\left(s_{ld}^{i},s_{ld}^{i+1}\right)$;
$t(s_{ld}^{i},s_{ld}^{i+1})$: travel time of the section $\left(s_{ld}^{i},s_{ld}^{i+1}\right)$;
[*T*_1_,*T*_2_]: operation period of the urban rail network;
*τ*_*l*_: minimum headway of line *l*;
*C*_*l*_: maximum capacity of a train for line *l*;
*S*_*F*_: set of transfer stations;
Δ*T*: the interval time;
*N*: total intervals of [*T*_1_,*T*_2_];
*t*_*k*_: the *k*th interval (*k* = 1,2,⋯,*N*);
*RS*: set of O-D pairs;
*q*_*rs*_(*t*_*k*_): O-D demand of pair (*r*,*s*);
*V*: node set of the urban rail transmission network (*V* = *S* ∪ *S*_*Ω*_);
*S*_*Ω*_: set of platforms;
*A*_qu_: set of queuing arcs which point from the station node to the platform node;
*A*_ar_: set of arriving arcs which point from the platform node to the station node;
*A*_tr_: set of transmission arcs;
*A*: set of arcs (*A* = *A*_qu_ ∪ *A*_ar_ ∪ *A*_tr_);
*a*: an arc in *A*;
*x*_*a*_(*t*_*k*_): number of passengers in the arc *a* ∈ *A* in *t*_*k*_;
$x_{a}^{qu}(t_{k})$: number of passengers along arc *a* ∈ *A*_qu_ in *t*_*k*_;
$x_{a}^{ar}(t_{k})$: number of passengers along arc *a* ∈ *A*_ar_ in *t*_*k*_;
$x_{a}^{tr}(t_{k})$: number of passengers along arc *a* ∈ *A*_tr_ in *t*_*k*_;
${\bar{x}}_{a}^{qu}(t_{k})$: number of passengers departing from arc *a* ∈ *A*_qu_ in *t*_*k*_;
*c*(*a*): cost of the arc *a* ∈ *A*;
*c*_qu_(*a*): cost of the arc *a* ∈ *A*_qu_;
*c*_ar_(*a*): cost of the arc *a* ∈ *A*_ar_;
*c*_tr_(*a*): cost of the arc *a* ∈ *A*_tr_;
*λ*: a parameter (0 < *λ* < 0.5);
*θ*: converted factor of time transforming to cost;
*k*(*s*^*u*^): number of time intervals transferring at *s*^*u*^;
*η*,*α*: cost parameters;
$n_{ld}^{i}$: number of cells in section $\left(s_{ld}^{i},s_{ld}^{i+1}\right)$;
Cell(*s*^*u*^): station cell of station *s*^*u*^;
Cell(*L*_*ld*_,*i*,*j*): the *j*th transmission cell in the *i*th section of directional line *L*_*ld*_;
*y*_*h*_(*s*^*u*^,*t*_*k*_): flow in station cell Cell(*s*^*u*^,*t*_*k*_);
*y*_*h*_(*L*_*ld*_,*i*,*j*,*t*_*k*_): flow in transmission cell Cell(*L*_*ld*_,*i*,*j*,*t*_*k*_);
*x*_*h*_(*s*^*u*^,*t*_*v*_), flow which arrives at station *s*^*u*^ in *t*_*v*_, and is detained at the station in *t*_*k*_;
*f*_*h*_(*L*_*ld*_,*i*,*end*,*t*_*k*_): outflow volume of tail cell Cell(*L*_*ld*_,*i*,*end*) in *t*_*k*_;
*m*(*L*_*ld*_,*i*): number of cells in the *i*th section of the directional line *L*_*ld*_;
$H(s_{ld}^{i+1},s_{ld}^{i+2},t_{k})$: set of destinations to which the shortest path from platform $s_{ld}^{i+1}$ passes through $s_{ld}^{i+2}$ in *t*_*k*_;
$H(s^{u},s_{ld}^{i},t_{k})$: set of destinations to which the shortest path from station *s*^*u*^ passes through $s_{ld}^{i}\in S(s^{u})$ in *t*_*k*_;
*C*_rem_(*L*_*ld*_,*i*,1,*t*_*k*_): surplus capacity of head cell Cell(*L*_*ld*_,*i*,1) in *t*_*k*_;
$M(s_{ld}^{i},t_{k})$: flow from Cell(*s*^*u*^) to $Cell(L_{ld},i,1),s_{ld}^{i}\in S(s^{u})$;
$s_{F}^{0}$: the first transfer station of a shortest path;
$s_{F}^{1}$: the last transfer station of a shortest path;
$p(s,s_{F}^{0})$: length of the first segment of the shortest path from the origin *s* to the first transfer station $s_{F}^{0}\in S$ along a directional line *L*^0^ ∈ *Ω*;
$p(s_{F}^{1},s^{u})$: length of the last segment of the shortest path from the last transfer station $s_{F}^{1}\in S$ to the destination *s*^*u*^ along a directional line *L*^1^ ∈ *Ω*;
*G*(*L*_*lU*_,*L*_*lD*_): network composed of directional lines *L*_*lU*_ and *L*_*lD*_;
$Z(s_{ld}^{i},t_{k})$: detained flow on the platform $s_{ld}^{i}$.

## Quantitative description of relevant concepts in the urban rail network

### Urban rail network

An urban rail network comprises a number of lines, and a line comprises a number of stations and sections. In urban rail system, trains are usually planned individually for each line. We assume that trains do not run across lines, and passengers can move across lines by transfer stations.

Given an urban rail network with *n* stations and *m* lines. The set of stations is denoted as *S* = {*s*^1^,*s*^2^,⋯,*s*^*n*^}. Urban rail lines are almost linear, and some complex urban rail lines, for example, annular lines or Y-style lines, can be decomposed into the linear lines, so we represent urban rail lines as linear. Each line includes two directional lines according to two opposite directions of operation, and the set of directional lines can be denoted as Ω = {*L*_1U_,*L*_1D_,*L*_2U_,*L*_2D_,⋯,*L*_*m*U_,*L*_*m*D_}, where *L*_*l*U_, *L*_*l*D_ denotes two directional lines of line *l*. We denote the direction variable as *d* ∈ {U,D}, and the directional line as *L*_*ld*_ ∈ Ω. For describing the queuing of passengers at the station, a station can be extended into many platforms for each directional line, and each platform only serves for a unique direction line, so a directional line can be described as a sequence of the platforms. We denote $s_{ld}^{i}$ as the *i*th platform of the directional line *L*_*ld*_, and *n*(*l*) as the number of the stations serving by line *l*. Then directional line *L*_*ld*_ can be denoted as a sequence of platforms $\left(s_{ld}^{1},s_{ld}^{2},\cdots ,s_{ld}^{n(l)}\right)$. Denote the set of platforms as $S_{\Omega }=\{s_{ld}^{i},i=1,2,\cdots ,n(l),L_{ld}\in \Omega \}$. A section can be denoted as a dual group $\left(s_{ld}^{i},s_{ld}^{i+1}\right)$. Therefore, the directional line *L*_*ld*_ can also be denoted as a sequence of sections $\left((s_{ld}^{1},s_{ld}^{2}),(s_{ld}^{2},s_{ld}^{3}),\cdots ,(s_{ld}^{n(l)-1},s_{ld}^{n(l)})\right)$, and $(s_{ld}^{i},s_{ld}^{i+1})\in L_{ld}$. Let *E* denote the set of sections. We define the stations passed through by two or more lines as the transfer stations, and let *S*_*F*_ ⊂ *S* denote the set of transfer stations. For convenience, we let ${\bar{s}}_{ld}^{i}\in S$ denote the corresponding station of platform $s_{ld}^{i}$, and *S*(*s*^*u*^) denote the set of platforms corresponding to station *s*^*u*^. For Section $\left(s_{ld}^{i},s_{ld}^{i+1}\right)$, we denote $d(s_{ld}^{i},s_{ld}^{i+1})$ as the mileage of the section $\left(s_{ld}^{i},s_{ld}^{i+1}\right)$, and $t(s_{ld}^{i},s_{ld}^{i+1})$ as the travel time of the section $\left(s_{ld}^{i},s_{ld}^{i+1}\right)$.

We denote the urban rail network as (*V*,*A*), where the node set *V* = *S* ∪ *S*_Ω_. Station nodes mainly describe the departure, arrival and transfer of passengers, while platform nodes mainly describe the passenger flow getting on/off trains, queuing and passing through stations. $A_{qu}=\left\{(s^{u},s_{ld}^{i}),s_{ld}^{i}\in S(s^{u}),s^{u}\in S\right\}$ denotes the set of queuing arcs which point from station nodes to platform nodes, $A_{ar}=\{(s_{ld}^{i},{\bar{s}}_{ld}^{i}),s_{ld}^{i}\in S_{\Omega }\}$ denotes the set of arriving arcs which point from the platform nodes to their corresponding station nodes and $A_{tr}=\{(s_{ld}^{i},s_{ld}^{i+1}),s_{ld}^{i},s_{ld}^{i+1}\in S_{\Omega }\}$ denotes the set of transmission arcs. Then, *A* = *A*_qu_ ∪ *A*_ar_ ∪ *A*_tr_.

**Fig 1:**
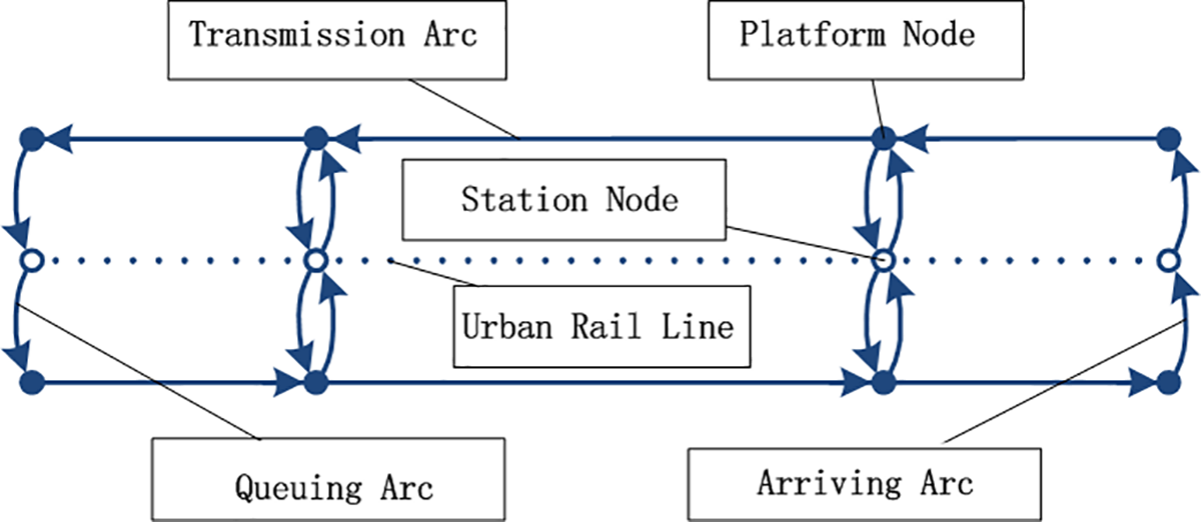
Illustration of the urban rail network with a single line.

**Fig 2:**
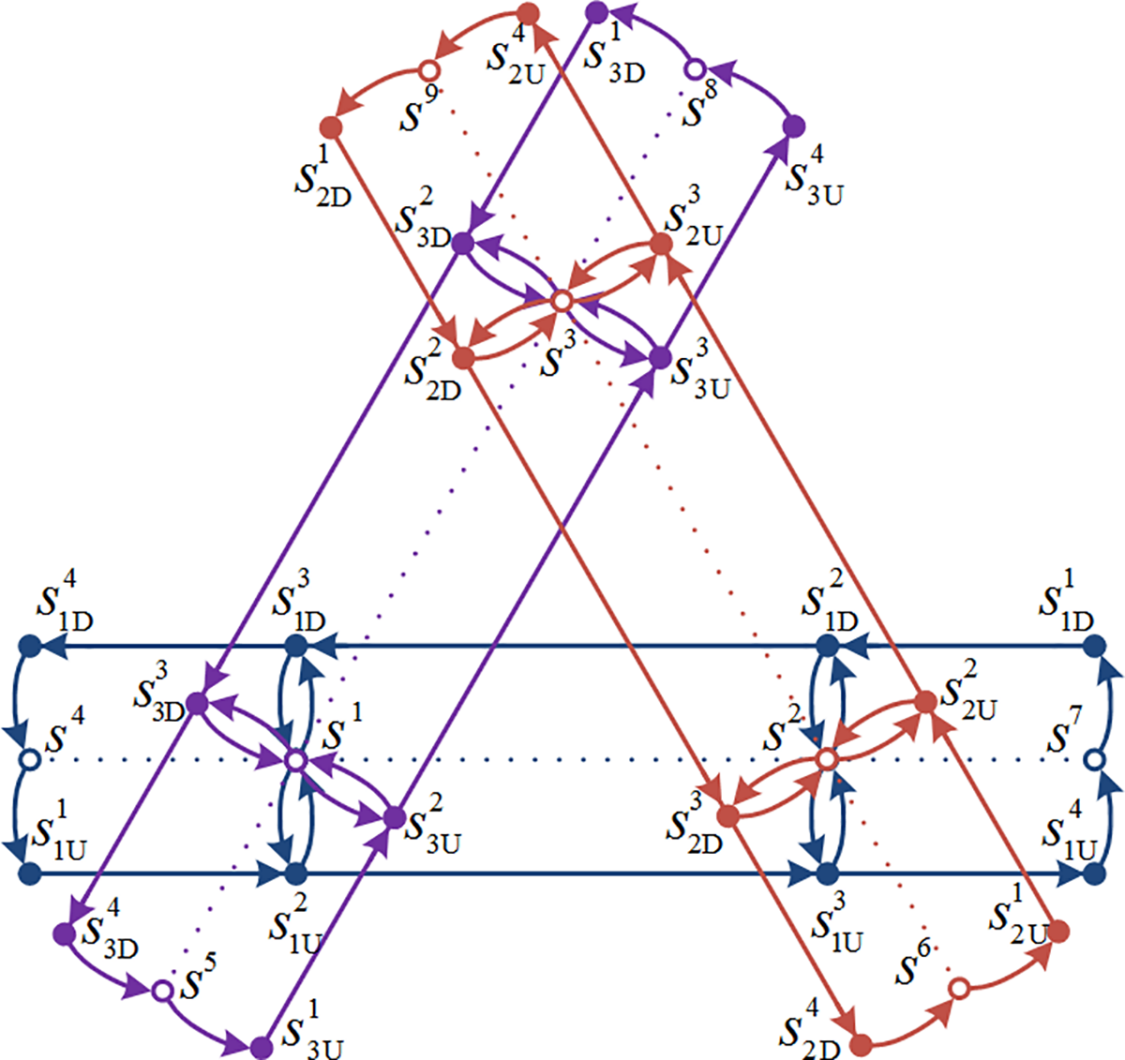
Illustration of the urban rail network with three lines.

The urban rail network with a single line is illustrated in **Fig 1**, where the dashed line represents an urban rail operating line and doesn’t belong to the urban rail network. The hollow nodes represent the station nodes and the solid nodes represent the platform nodes. The upside part in **Fig 1** shows one directional line, while the downside part shows the other directional line. **Fig 2** shows an urban rail network with three lines. In **Fig 2**, ${\bar{s}}_{1U}^{2}={\bar{s}}_{1D}^{3}={\bar{s}}_{3U}^{2}={\bar{s}}_{3D}^{3}=s^{1}$, and $S(s^{1})=\{s_{1U}^{2},s_{1D}^{3},s_{3U}^{2},s_{3D}^{3}\}$.

The above described network structure does not include the annular lines or Y-style lines (a Y-style line is a structure where two separate lines merge into one at a station), but it is easy to transform them into linear lines. For example, if $s_{ld}^{1}$ and $s_{ld}^{n(l)}$ are viewed as the same platform, the above network can describe the annular lines. For simplicity, we do not give specialized descriptions for lines with those special structures. Moreover, the above network does not describe the train stopping process in order to decrease the scale of the urban rail network.

### Constrained continuous transmission

To avoid the restrictions of the schedules, the concept of continuous transmission is introduced. Continuous transmission means that each rail line can serve passengers at any time and passenger’s traveling is not restricted by the train’s schedule times, just like the road transportation.

To account for the urban rail’s capacity constraint, continuous transmission has a capacity constraint, which means that the passenger transmission intensity of directional line *L*_*ld*_ at any time cannot exceed the transmission capacity *C*_*l*_/*τ*_*l*_, where *C*_*l*_ is the capacity of each trains in line *l*, and *τ*_*l*_ is the minimum headway of line *l*. We denote [*T*_1_,*T*_2_] as the operation period of the urban rail network. Divide [*T*_1_,*T*_2_] into *N* equal intervals by the interval Δ*T*, and denote *t*_*k*_, *k* = 1,2,⋯,*N*, as the *k*th interval. For each time interval, its transmission capacity is Δ*T C*_*l*_/*τ*_*l*_, *L*_*ld*_ ∈ Ω.

The benefit of the introduction of continuous transmission is that the space-time flow distribution obtained by DAURTN is not restricted by urban rail schedule, and can be used to evaluate and optimize the space-time resource allocation, for example, the schedule, the rolling stock circulation, and so on.

### Priority principle

In each time interval, passenger flow cannot exceed the transmission capacity. When the flow exceeds the transmission capacity, only part of passengers can be transmitted during the current time interval, and surplus passengers have to wait at the station. According to the travel behavior of urban rail transit, the passengers’ choices for different O–D pairs with capacity constrains must obey the following priority principles:

Space priority principle: the flows of upstream stations along the directional line have priority over those of downstream stations to occupy capacities.

This principle is from the papers of [9–11] and it means when loading a vehicle, on-board passengers continuing to the next stop had priority. Under the space priority principle, passengers at upstream stations will not reserve capacity for the waiting passengers at downstream stations.

First-come-first-serve (FCFS) principle: passengers arriving earlier have priority over those arriving later to obtain service at a station.

Under the first-come-first-serve principle, the limited transmission capacity will be provided for passengers in the order of the batches arriving at the stations, and for one batch of passengers with different destinations, the method of equal proportional competition is used to determine the flow of departing passengers [10].

### Demands and costs

The origins and destinations of all O–D pairs belong to the station node set *S*. We denote *RS* as the set of O–D pairs, and *q*_*rs*_(*t*_*k*_), (*r*,*s*) ∈ *RS*, 1 ≤ *k* ≤ *N* as O–D demands at time interval *t*_*k*_. In this study, it is assumed that passengers follow the instantaneous dynamic route choice principle [22], i.e., passengers choose the minimal cost routes under the currently time interval. Passenger flow reaches the instantaneous dynamic user optimal state that for each O-D pair at each decision node at each time interval, the instantaneous travel costs for all routes that are being used equal the minimal instantaneous route travel time [22].

In urban rail network (*V*,*A*), for any interval *t*_*k*_, 1 ≤ *k* ≤ *N*, denote *x*_*a*_(*t*_*k*_) as the flow on arc *a* ∈ *A* in *t*_*k*_. When *a* ∈ *A*_qu_,*A*_ar_ or *A*_tr_, use $x_{a}^{qu}(t_{k}),x_{a}^{ar}(t_{k}),x_{a}^{tr}(t_{k})$ to replace *x*_*a*_(*t*_*k*_) respectively. Especially for *a* ∈ *A*_tr_, $x_{a}^{tr}(t_{k})$ is equal to the cumulating flow of differences between inflow and outflow from time interval *t*_1_ to *t*_*k*_, with the concept of the continuous transmission. This method of calculation is similar to that for road link flow in dynamic route choice models [22]. We denote ${\bar{x}}_{a}^{qu}(t_{k})$ as the flow departing and transmitted from arc *a* ∈ *A*_qu_ in *t*_*k*_. The cost of arc *a* is denoted as *c*(*a*), *a* ∈ *A*. When *a* ∈ *A*_qu_,*A*_ar_ or *A*_tr_, we use *c*_qu_(*a*),*c*_ar_(*a*) or *c*_tr_(*a*) to replace *c*(*a*) respectively.

Based on the continuous transmission and instantaneous dynamic route choice principle, we calculate the queuing time by the flow state in the current time interval, i.e., the total queuing flow $x_{a}^{qu}(t_{k})$ and the transmitted passenger flow ${\bar{x}}_{a}^{qu}(t_{k})$ at the platform of the queuing arc *a* in the current time interval. Thus, the number of time intervals queuing at platform is $x_{a}^{qu}(t_{k})/{\bar{x}}_{a}^{qu}(t_{k})$, and the queuing time is $\Delta Tx_{a}^{qu}(t_{k})/{\bar{x}}_{a}^{qu}(t_{k})$. However, ${\bar{x}}_{a}^{qu}(t_{k})$ may tend to or be equal to zero, which will result in too large congestion cost, so we assume that denominator has a lower limit, which is set to be *λC*_*l*_, where *λ* is a parameter. Therefore, the queuing time estimated by passengers is

$$\Delta Tx_{a}^{qu}(t_{k})/\max\left\{{\bar{x}}_{a}^{qu}(t_{k}),\lambda C_{l}\right\}$$ (1)

and then, the queuing cost can be expressed as

$$c_{qu}(a)=\theta \Delta Tx_{a}^{qu}(t_{k})/\max\left\{{\bar{x}}_{a}^{qu}(t_{k}),\lambda C_{l}\right\}$$ (2)

where *θ* is the converted factor for transforming time to cost.

For an arriving arc *a* ∈ *A*_ar_,*c*_ar_(*a*) is the cost of the average transfer walking time, and needed to be expressed as a multiple of Δ*T*. We denote *s*_*a*_ as the arrival station of arc *a*. Assuming that the average transfer time at *s*_*a*_ is a constant, denote it as const(*s*_*a*_), then

$$c_{ar}(a)=\theta const(s_{a})$$ (3)

Denote the number of time intervals for transferring at station *s*_*a*_ as *k*(*s*_*a*_), then

$$k(s_{a})=\lceil const(s_{a})/\Delta T\rceil$$ (4)

where ⌈*x*⌉ is the function of minimum integer no less than *x*.

For a transmission arc *a* ∈ *A*_tr_, the cost *c*_tr_(*a*) is the sum of section travel cost and congestion cost, namely

$$c_{tr}(a)=\theta t(s_{ld}^{i},s_{ld}^{i+1})+g(x_{a}^{tr}(t_{k}))$$ (5)

where $g(x_{a}^{tr}(t_{k}))$ is the congestion cost.

For a transmission arc *a* ∈ *A*_tr_, the total flow on arc *a* at time interval *t*_*k*_ is $x_{a}^{tr}(t_{k})$, and the total capacity of arc *a* is denoted as *C*_*a*_, so the congestion cost $g(x_{a}^{tr}(t_{k}))$ of arc *a* can be expressed as follows:

$$g(x_{a}^{tr}(t_{k}))=\eta t(s_{ld}^{i},s_{ld}^{i+1}){\left[\frac{x_{a}^{tr}(t_{k})}{C_{a}}\right]}^{\alpha }$$ (6)

which is similar to the power form used in BPR functions and the congestion functions in the papers of [10] and [47], and where *η* and *α* are cost parameters and *η*,*α* > 0. The congestion cost function is increasing with travel time and passenger flow volume. With the concept of constrained continuous transmission, the transmission arc *a* can be regarded as a train with the length $\left(s_{ld}^{i},s_{ld}^{i+1}\right)$, of which the capacity per length unit is *C*_*l*_/*τ*_*l*_, so the capacity of the transmission arc *a* ∈ *A*_tr_ is calculated as

$$C_{a}=t(s_{ld}^{i},s_{ld}^{i+1})*C_{l}/\tau_{l}$$ (7)

The congestion cost $g(x_{a}^{tr}(t_{k}))$ can be obtained by substituting Eq (7) into Eq (6):

$$\begin{array}{l} g(x_{a}^{tr}(t_{k}))=\eta t(s_{ld}^{i},s_{ld}^{i+1}){\left[\frac{x_{a}^{tr}(t_{k})}{t(s_{ld}^{i},s_{ld}^{i+1})*C_{l}/\tau_{l}}\right]}^{\alpha }\\ \;=\eta {{t(s_{ld}^{i},s_{ld}^{i+1})}^{1-\alpha }(\tau_{l}x_{a}(t_{k})/C_{l})}^{\alpha } \end{array}$$ (8)

In Eq (8), the travel time $t(s_{ld}^{i},s_{ld}^{i+1})$ is fixed and determined, and only $x_{a}^{tr}(t_{k})$ is variable. Hence, when *η*,*α* > 0, the calculation of congestion is feasible and the congestion influences in the travel choice of passengers.

## Cell transmission model for DAURTN

### Cell transmission mechanism

To solve the DAURTN based on the continuous transmission, we build the CTM for the urban rail network (*V*,*A*). For describing the CTM, the cell transmission network is constructed based on cell from the network as follows.

We define each section as a cell chain, and each station as a station cell. For any section $\left(s_{ld}^{i},s_{ld}^{i+1}\right)$, as travel time $t(s_{ld}^{i},s_{ld}^{i+1})$ is fixed, we divide the section into several transmission cells by Δ*T*. The transmission cells of one section compose a cell chain, and passengers flows can be transmitted forward between the transmission cells. Note that travel time $t(s_{ld}^{i},s_{ld}^{i+1})$ may not be exactly divided by Δ*T*, so the time length of the tail cell can be equal to or exceed Δ*T*. The number of cells divided is $n_{ld}^{i}=\lfloor t(s_{ld}^{i},s_{ld}^{i+1})/\Delta T\rfloor$, where ⌊*x*⌋ is a function of the maximum integer no larger than *x*. Therefore, we denote Cell(*L*_*ld*_,*i*,*j*) as the *j*th cell in the cell chain of section $\left(s_{ld}^{i},s_{ld}^{i+1}\right)$. Denote *m*(*L*_*ld*_,*i*) as the number of cells in the *i*th cell chain, and for simplifying the notation, denote Cell(*L*_*ld*_,*i*,*end*) the last cell in the cell chain of section $\left(s_{ld}^{i},s_{ld}^{i+1}\right)$. Denote *y*_*h*_(*L*_*ld*_,*i*,*j*,*t*_*k*_) as the flow of Cell(*L*_*ld*_,*i*,*j*) traveling to destination *s*^*h*^ in *t*_*k*_. We also denote Cell(*s*^*u*^) as the station cell of *s*^*u*^, and *y*_*h*_(*s*^*u*^,*t*_*k*_) as the flow of Cell(*s*^*u*^) traveling to destination *s*^*h*^ in *t*_*k*_.

**Fig 3:**
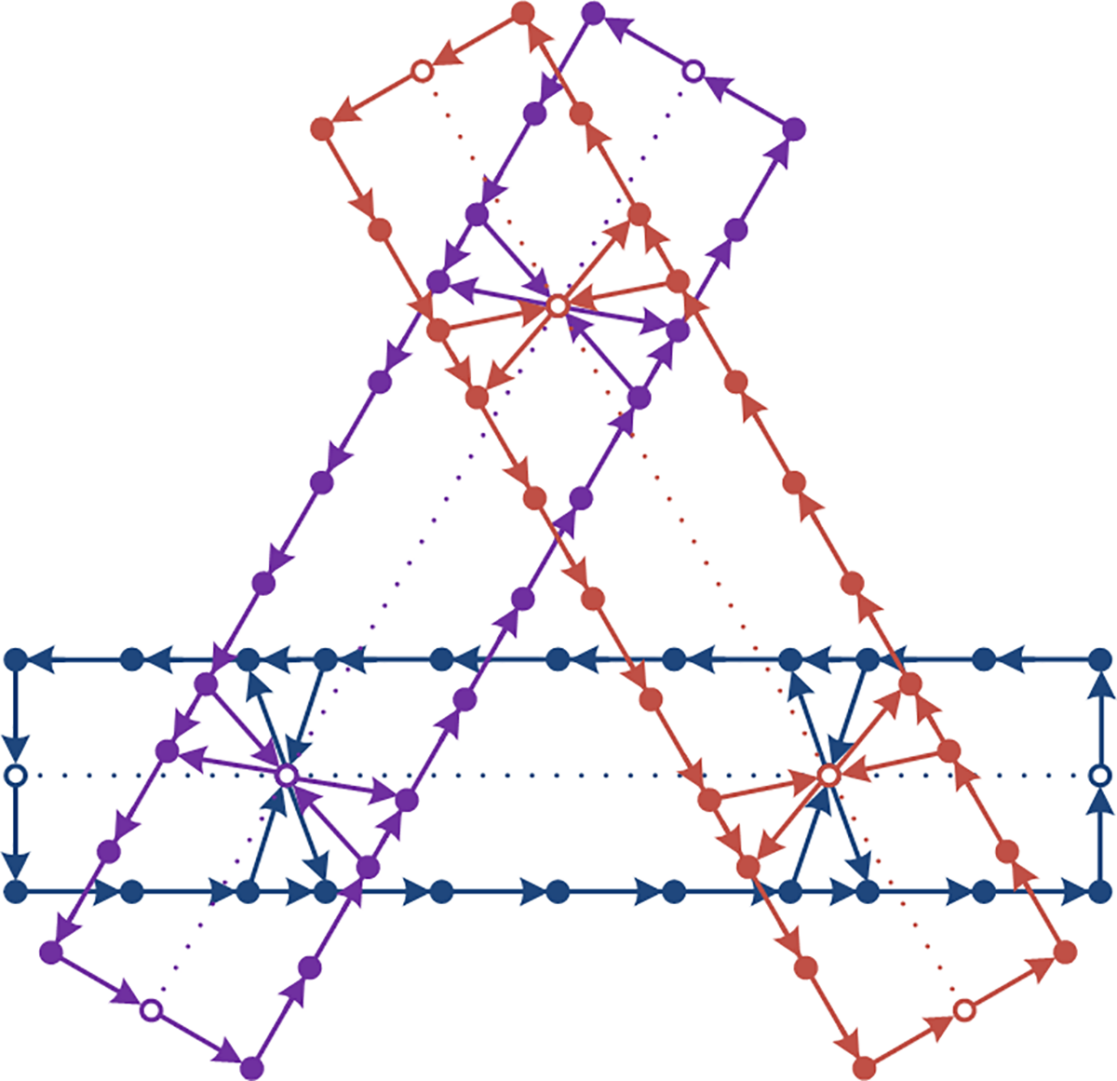
Illustration of the cell transmission network.

The transmission relationship between cells is illustrated in **Fig 3**, where a hollow node represents a station cell, a solid node represents a transmission cell, a hollow rectangle represents the corresponding cell chain of a section, and the arrows represent transmission directions. In each time interval, passengers follow the instantaneous dynamic route choice principle [22] and are transmitted between cells. Transmission mechanism between cells is designed and transmission processes of flows between the cells are classified into 4 groups:

$$\begin{array}{l} \mathrm{Cell}\left(L_{ld},i,end\right)⟹\mathrm{Cell}\left(L_{ld},i+1,1\right),\;\forall \;i\leq n\left(l\right)-1,L_{ld};\\ \mathrm{Cell}\left(L_{ld},i,end\right)⟹\mathrm{Cell}\left({\bar{s}}_{ld}^{i+1}\right),\;\forall \;i\leq n\left(l\right)-1,L_{ld};\\ \mathrm{Cell}\left(L_{ld},i,j\right)⟹\mathrm{Cell}\left(L_{ld},i,j+1\right),\;\forall j\leq m\left(L_{ld},i\right)-1,i,L_{ld};\\ \mathrm{Cell}\left(s^{u}\right)⟹\mathrm{Cell}\left(L_{ld},i,1\right),\;s_{ld}^{i}\in S\left(s^{u}\right),\forall u,i,L_{ld}. \end{array}$$

where ‘⟹’ means the transmission process of flow from the left cell to the right cell.

The flows of station cells and transmission cells at the initial interval *t*_0_ are 0. In *t*_*k*_(*k* ≥ 1), the O–D demand {*q*_*uh*_(*t*_*k*_)|*s*^*h*^ ∈ *S*} inflows into station cell Cell(*s*^*u*^). Therefore, the initial value of the variables are *y*_*h*_(*s*^*u*^,*t*_0_) = 0, *y*_*h*_(*s*^*u*^,*t*_*k*_) = *q*_*uh*_(*t*_*k*_), *y*_*h*_(*L*_*ld*_,*i*,*j*,*t*_*k*_) = 0, ∀*h*,*u*,*i*,*j*,*L*_*ld*_,*k* ≥ 1.

Next, we analyze the transmission mechanism in 3 steps.

Step 1: The transmission processes Cell(*L*_*ld*_,*i*,*end*) ⟹ Cell(*L*_*ld*_,*i* + 1,1), and $Cell(L_{ld},i,end)⟹Cell({\bar{s}}_{ld}^{i+1})$

As the length of time in the tail cell Cell(*L*_*ld*_,*i*,*end*) is equals to or greater than Δ*T*, only a certain proportion of flow can outflow, and the proportion is $\Delta T/\left[t(s_{ld}^{i},s_{ld}^{i+1})-(n_{ld}^{i}-1)\Delta T\right]$. Thus, the outflow of Cell(*L*_*ld*_,*i*,*end*) in interval *t*_*k*_ is

$$f_{h}(L_{ld},i,end,t_{k})=y_{h}(L_{ld},i,j,t_{k-1})\Delta T/\left[t(s_{ld}^{i},s_{ld}^{i+1})-(n_{ld}^{i}-1)\Delta T\right],\forall h$$ (9)

Then we can obtain the detained flow of the tail cell

$$y_{h}(L_{ld},i,end,t_{k})\leftarrow y_{h}(L_{ld},i,end,t_{k-1})-f_{h}(L_{ld},i,end,t_{k}),\forall h$$ (10)

where ‘←’ denotes that the value of the right variable is assigned to the left variable.

According to the space priority principle, the outflow *f*_*h*_(*L*_*ld*_,*i*,*end*,*t*_*k*_) of tail cell Cell(*L*_*ld*_,*i*,*end*) has only two choices, i.e., Cell(*L*_*ld*_,*i* + 1,1) or $Cell({\bar{s}}_{ld}^{i+1})$, and the transmission choice is determined by the instantaneous dynamic route choice principle [22], i.e., the shortest path from platform $s_{ld}^{i+1}$ to destination station *s*^*h*^ at the current time interval in network (*V*,*A*). If the shortest path passes through $s_{ld}^{i+2}$, then flow *f*_*h*_(*L*_*ld*_,*i*,*end*,*t*_*k*_) is transmitted into cell Cell(*L*_*ld*_,*i* + 1,1); otherwise, it is transmitted to station cell $Cell({\bar{s}}_{ld}^{i+1})$. The above transmission choice is similar to the all-or-nothing assignment, i.e., the flows follow the shortest path.

We denote the set of destinations to which the shortest path from platform $s_{ld}^{i+1}$ passes through $s_{ld}^{i+2}$ as

$$H(s_{ld}^{i+1},s_{ld}^{i+2},t_{k})=\{s^{h}\in S|in\;t_{k},\;the\;shortest\;path\;from\;s_{ld}^{i+1}\;to\;s^{h}\;passes\;through\;s_{ld}^{i+2}\}$$ (11)

When $s^{h}\in H(s_{ld}^{i+1},s_{ld}^{i+2},t_{k})$, the flow *f*_*h*_(*L*_*ld*_,*i*,*end*,*t*_*k*_) is transmitted from Cell(*L*_*ld*_,*i*,*end*) to Cell(*L*_*ld*_,*i* + 1,1). Thus,

$$y_{h}(L_{ld},i+1,1,t_{k})\leftarrow y_{h}(L_{ld},i+1,1,t_{k})+f_{h}(L_{ld},i,end,t_{k}),s^{h}\in H(s_{ld}^{i+1},s_{ld}^{i+2},t_{k})$$ (12)

When $s^{h}\notin H(s_{ld}^{i+1},s_{ld}^{i+2},t_{k}),s^{h}\neq {\bar{s}}_{ld}^{i+1}$, the flow *f*_*h*_(*L*_*ld*_,*i*,*end*,*t*_*k*_) is transmitted from Cell(*L*_*ld*_,*i*,*end*) to $Cell({\bar{s}}_{ld}^{i+1})$. Note that the average transfer time at station ${\bar{s}}_{ld}^{i+1}$ is $k({\bar{s}}_{ld}^{i+1})$, so

$$y_{h}\left({\bar{s}}_{ld}^{i+1},t_{k+k({\bar{s}}_{ld}^{i+1})}\right)\leftarrow y_{h}\left({\bar{s}}_{ld}^{i+1},t_{k+k({\bar{s}}_{ld}^{i+1})}\right)+f_{h}(L_{ld},i,end,t_{k}),s^{h}\notin H(s_{ld}^{i+1},s_{ld}^{i+2},t_{k}),s^{h}\neq {\bar{s}}_{ld}^{i+1}$$ (13)

In order to realize the FCFS in transmission mechanism, we introduce a variable *x*_*h*_(*s*^*u*^,*t*_*v*_), 1 ≤ *v* ≤ *N*, which represents the flows arriving at station *s*^*u*^ in *t*_*v*_ and detained at the station in *t*_*k*_.

$$x_{h}\left({\bar{s}}_{ld}^{i+1},t_{k+k({\bar{s}}_{ld}^{i+1})}\right)\leftarrow x_{h}\left({\bar{s}}_{ld}^{i+1},t_{k+k({\bar{s}}_{ld}^{i+1})}\right)+y_{h}\left({\bar{s}}_{ld}^{i+1},t_{k+k({\bar{s}}_{ld}^{i+1})}\right)$$ (14)

Step 2: The transmission process Cell(*L*_*ld*_,*i*,*j*) ⟹ Cell(*L*_*ld*_,*i*,*j* + 1)

After Step 1, in the tail cell of the cell chain, there may be some detained flows, and then the flow of the tail cell equals to the detained flows plus the flows from Cell(*L*_*ld*_,*i*,*end* − 1), so

$$y_{h}(L_{ld},i,end,t_{k})\leftarrow y_{h}(L_{ld},i,end,t_{k})+y_{h}(L_{ld},i,end-1,t_{k-1})$$ (15)

For other cells in the chain, it is only need to move flows from the forward cell to the backward cell in the chain, that is,

$$y_{h}(L_{ld},i,j+1,t_{k})\leftarrow y_{h}(L_{ld},i,j,t_{k-1}),\;1\leq j\leq m(L_{ld},i)-2$$ (16)

Step 3: The transmission process $Cell(s^{u})⟹Cell(L_{ld},i,1),s_{ld}^{i}\in S(s^{u})$

According to the principle of the space priority, flows from Cell(*L*_*ld*_,*i* − 1,*end*) are transmitted to Cell(*L*_*ld*_,*i*,1) and occupy the capacity of Cell(*L*_*ld*_,*i*,1) with priority. Thus, the surplus capacity of Cell(*L*_*ld*_,*i*,1) in *t*_*k*_ is

$$C_{rem}(L_{ld},i,1,t_{k})=\Delta T\;C_{l}/\tau_{l}-y_{h}(L_{ld},i,1,t_{k})$$ (17)

The flows in *t*_*k*_, which are queuing at station *s*^*u*^ and head to Cell(*L*_*ld*_,*i*,1), have to compete for the surplus capacity with the FSFC principle.

In order to determine the queuing flow, passengers at station cell Cell(*s*^*u*^) first determine which platform to queue. Similar to the method in Step 1, passengers determined the platform by the shortest path from station *s*^*u*^ to destination station *s*^*h*^ at the current time interval in network (*V*,*A*). If the shortest path passes through $s_{ld}^{i}\in S(s^{u})$, then flows traveling to destination station *s*^*h*^ queue on platform $s_{ld}^{i}$. We denote the set of destinations to which the shortest path from station *s*^*u*^ passes through $s_{ld}^{i}\in S(s^{u})$ as

$$H(s^{u},s_{ld}^{i},t_{k})=\{s^{h}\in S|in\;t_{k},\;the\;shortest\;path\;from\;s^{u}\;to\;s^{h}\;passes\;through\;s_{ld}^{i}\in S(s^{u})\}$$ (18)

Thus, the flow competing for the surplus capacity is $y_{h}(s^{u},t_{k}),\;s^{h}\in H(s^{u},s_{ld}^{i},t_{k}),s_{ld}^{i}\in S(s^{u})$.

If $\sum_{s^{h}\in H(s^{u},s_{ld}^{i},t_{k})}y_{h}(s^{u},t_{k})\leq C_{rem}(L_{ld},i,1,t_{k})$, then

$$\left\{ \begin{array}{l} y_{h}(L_{ld},i,1,t_{k})\leftarrow y_{h}(L_{ld},i,1,t_{k})+y_{h}(s^{u},t_{k}),\;s^{h}\in H(s^{u},s_{ld}^{i},t_{k})\\ x_{h}(s^{u},t_{v})\leftarrow 0,\;v<k,s^{h}\in H(s^{u},s_{ld}^{i},t_{k}) \end{array}\right.$$ (19)

If $\sum_{s^{h}\in H(s^{u},s_{ld}^{i},t_{k})}y_{h}(s^{u},t_{k})>C_{rem}(L_{ld},i,1,t_{k})$, which means the surplus capacity is insufficient, then there exists $\bar{k}<k$, and it makes that

$$\sum_{s^{h}\in H(s^{u},s_{ld}^{i},t_{k})}\sum_{v<\bar{k}}x_{h}(s^{u},t_{v})\leq C_{rem}(L_{ld},i,1,t_{k})<\sum_{s^{h}\in H(s^{u},s_{ld}^{i},t_{k})}\sum_{v\leq \bar{k}}x_{h}(s^{u},t_{v})$$

According to the FCFS principle, the flow $\sum_{v<\bar{k}}x_{h}(s^{u},t_{v})$ traveling to each destination station $s^{h}\in H(s^{u},s_{ld}^{i},t_{k})$ can be transmitted, i.e.,

$$\left\{ \begin{array}{l} y_{h}(L_{ld},i,1,t_{k})\leftarrow y_{h}(L_{ld},i,1,t_{k})+\sum_{v<\bar{k}}x_{h}(s^{u},t_{v}),\;s^{h}\in H(s^{u},s_{ld}^{i},t_{k})\\ x_{h}(s^{u},t_{v})\leftarrow 0,\;v<\bar{k},s^{h}\in H(s^{u},s_{ld}^{i},t_{k}) \end{array}\right.$$ (20)

and a portion of $\sum_{s^{h}\in H(s^{u},s_{ld}^{i},t_{k})}x_{h}(s^{u},t_{\bar{k}})$ can also be transmitted. According to the equal proportion principle, the proportion of flows transmitted can be calculated by

$$\alpha =\left[C_{rem}(L_{ld},i,1,t_{k})-\sum_{s^{h}\in H(s^{u},s_{ld}^{i},t_{k})}\sum_{v<\bar{k}}x_{h}(s^{u},t_{v})\right]/\sum_{s^{h}\in H(s^{u},s_{ld}^{i},t_{k})}x_{h}(s^{u},t_{\bar{k}})$$ (21)

Then

$$\left\{ \begin{array}{l} y_{h}(L_{ld},i,1,t_{k})\leftarrow y_{h}(L_{ld},i,1,t_{k})+\alpha x_{h}(s^{u},t_{\bar{k}})\\ x_{h}(s^{u},t_{\bar{k}})\leftarrow (1-\alpha )x_{h}(s^{u},t_{\bar{k}}) \end{array}\right.,\;s^{h}\in H(s^{u},s_{ld}^{i},t_{k})$$ (22)

After the above processes, the flow of each cell make a choice by the shortest paths and are all transmitted to the next cell. But the cost of each arc will be changed with the variable flow, so the method of successive average (MSA) is adopted to reach the instantaneous dynamic user optimal state in each time interval. The variables in the above model are updated in MSA.

### An efficient method for solving the shortest path

In the CTM, it is needed to solve the shortest path in *t*_*k*_ from s ∈ *S* ∪ *S*_Ω_ to *s*^*u*^ ∈ *S* in network (*V*,*A*). We design a fast method for solving the shortest path as follows.

If the shortest path from *s* ∈ *S* ∪ *S*_Ω_ to *s*^*u*^ ∈ *S* passes through several transfer stations, then the shortest path can be divided into three segments at most. The first segment of the shortest path is from origin *s* to the first transfer station $s_{F}^{0}\in S$, and its length is denoted as $p(s,s_{F}^{0})$. The last segment of the shortest path is from the last transfer station $s_{F}^{1}\in S$ to destination *s*^*u*^, and its length is $p(s_{F}^{1},s^{u})$. As long as we solve the length of the shortest path between any two transfer stations $p(s_{F}^{0},s_{F}^{1})$, we can obtain the cost of the shortest paths in three cases as follows:

$$p\left(s,s^{u}\right)=\left\{ \begin{array}{l} \min\left\{p\left(s,s_{F}^{0}\right)+p\left(s_{F}^{0},s_{F}^{1}\right)+p\left(s_{F}^{1},s^{u}\right)|s_{F}^{0},s_{F}^{1}\in S_{F},\exists L^{0},L^{1}\in \Omega :s,s_{F}^{0}\in L^{0},s_{F}^{1},s^{u}\in L^{1}\right\},\;s,s^{u}\notin S_{F}\\ \min\left\{p\left(s,s_{F}^{1}\right)+p\left(s_{F}^{1},s^{u}\right)|s_{F}^{1}\in S_{F},\exists L^{1}\in \Omega :s^{u},s_{F}^{1}\in L^{1}\right\},\;s\in S_{F},s^{u}\notin S_{F}\\ \min\left\{p\left(s,s_{F}^{0}\right)+p\left(s_{F}^{0},s^{u}\right)|s_{F}^{0}\in S_{F},\exists L^{0}\in \Omega :s,s_{F}^{0}\in L^{0}\right\},\;s\notin S_{F},s^{u}\in S_{F} \end{array}\right.$$ (23)

If the shortest path from *s* ∈ *S* ∪ *S*_Ω_ to *s*^*u*^ ∈ *S* does not pass through any transfer station, then it will only use one line and can be solved easily.

The above analysis indicates that the solving method for the shortest path from *s* ∈ *S* ∪ *S*_Ω_ to *s*^*u*^ ∈ *S* can be decomposed into 3 steps.

Step 1: calculate the shortest path from *s* ∈ *S* ∪ *S*_Ω_ to *s*^*u*^ ∈ *S* in each network *G*(*L*_*l*U_,*L*_*l*D_), which composed of a pair of opposite directional lines *L*_*l*U_,*L*_*l*D_ shown in **Fig 1**.

Step 2: calculate the shortest path between each two transfer stations in the network.

Step 3: calculate all the shortest paths from *s* ∈ *S* ∪ *S*_Ω_ to *s*^*u*^ ∈ *S*.

In step 1, for any destination $s^{u}={\bar{s}}_{lU}^{v}\in \{{\bar{s}}_{lU}^{i},i=1,2,\cdots ,n(l)\}$, we can structure two subsets of nodes bounded by node $s_{lU}^{v}$, i.e. $\left\{s_{lU}^{i},s_{lD}^{\left(n(l)-i+1\right)},{\bar{s}}_{lU}^{i},i=1,2,\cdots ,v\right\}$ and $\left\{s_{lU}^{i},s_{lD}^{\left(n(l)-i+1\right)},{\bar{s}}_{lU}^{i},i=v,v+1,\cdots ,n(l)\right\}$, which can form two generated sub-networks of *G*(*L*_*l*U_,*L*_*l*D_). Obviously, the shortest paths from other nodes to *s*^*u*^ in the two sub-networks are equal to solving the shortest paths in *G*(*L*_*l*U_,*L*_*l*D_). In the former sub-network, there are three cases.

Case 1: solve the shortest paths from nodes $s_{lU}^{i},i=1,2,\cdots ,v$ to *s*^*u*^ along the directional line *L*_*l*U_;

Case 2: solve the shortest paths from $s_{lD}^{n(l)}$ and ${\bar{s}}_{lU}^{1}$ to *s*^*u*^ passing through $s_{lU}^{1}$;

Case 3: solve the shortest paths from $s_{lD}^{n(l)-i}$ and ${\bar{s}}_{lU}^{i}$ to *s*^*u*^ containing the shortest path from $s_{lD}^{n(l)-i+1}$ to *s*^*u*^, or from $s_{lU}^{i}$ to *s*^*u*^.

Thus we can solve the shortest paths from $s_{lD}^{n(l)-i}$ and ${\bar{s}}_{lU}^{i}$ to *s*^*u*^ in the order of *i* = 2,3,⋯,*v*. In the latter sub-network, the solving method is similar. Therefore, the amount of calculation for the shortest paths from *s* ∈ *S* ∪ *S*_Ω_ to *s*^*u*^ ∈ *S* in network *G*(*L*_*l*U_,*L*_*l*D_) is only *O*(*n*(*l*)^2^), and the sum of calculation for the shortest paths of the whole *m* lines is $O(\sum_{l=1}^{m}{n(l)}^{2})$.

The method for solving the shortest paths for an annular line only needs to make some supplements based on the above method for linear lines. For any destination *s*^*u*^, we can divide the annular line into a linear line by *s*^*u*^, and there are only two ways of dividing. Then we can adopt the above method to solve the shortest paths from *s* ∈ *S* ∪ *S*_Ω_ to *s*^*u*^ for each way of dividing, and the shorter one between them is the shortest path. For a Y-style line, the similar method for solving the shortest paths is feasible.

We have obtained the length of shortest path between each two transfer stations in each line in step 1. In step 2, we use the Floyd–Warshall algorithm to solve the shortest path between any two transfer stations, and the computational complexity is *O*(|*S*_*F*_|^3^).

In step 3, we can solve the shortest paths from *s* ∈ *S* ∪ *S*_Ω_ to *s*^*u*^ ∈ *S* by formula (23), and the computational complexity is $O(|S|∙\sum_{l=1}^{m}n(l))$.

Therefore, the computational complexity of the shortest path solving algorithm in the urban rail transmission network is $O(|S|∙\sum_{l=1}^{m}n(l)+{\left|S_{F}\right|}^{3})$, which is less than $O({\left(\sum_{l=1}^{m}n(l)\right)}^{3})$ of the classical methods, i.e., Dijkstra algorithm and Floyd algorithm.

The urban rail network in Beijing composes 15 operating lines and 231 stations until July 2014. There are only 40 transfer stations, which are far less than other stations. Thus, the solving algorithm of the shortest path is effective for the real urban rail network. We test our method and Floyd algorithm for solving the shortest paths of Beijing urban rail network. Using the Matlab(R2010b) to program, the shortest path problem is calculated for 100 times, and we record the CPU times for the two methods. The average CPU time of Floyd algorithm is 10.31s, while the average CPU time of our method is 1.06s, with the computer (IntelCore 2.90GHz, 8GB RAM).

### Evaluation indexes of passenger flow

The CTM can generate many important evaluation indexes, including time-varying section flow, operating line circulation volume, the detained passenger volume on the platform, and the queuing length on the platform.

As the flow $x_{a}^{tr}(t_{k})$ for *a* ∈ *A*_tr_ is equal to the sum of the flows of all cells in this cell chain, we defined that the time-varying section flow means the outflow of the last cell in each time interval, which means passenger flow transmitted by the line section in each time interval and can reflect the capacity constraints in CTM. Thus, the section flow is

$$\sum_{h=1}^{n}f_{h}(L_{ld},i,end,t_{k}),\;\forall i,L_{ld},k$$ (24)

It is obvious that the time-varying section flow varies with Δ*T*, i.e., if Δ*T* becomes longer, then the time-varying section flow for each time interval is larger.

We can obtain the circulation volume of each directional line, which means the sum of travel mileages of passengers on each directional line, that is,

$$\sum_{k=1}^{N}\sum_{d\in \{U,D\}}\sum_{i=1}^{n(l)-1}\left[d(s_{ld}^{i},s_{ld}^{i+1})\sum_{h=1}^{n}f_{h}(L_{ld},i,end,t_{k})\right],\;\forall l$$ (25)

and its unit is person kilometer. The circulation volume of the whole network can be obtained by summing the circulation volumes of all directional lines.

In CTM, we can calculate the detained flow volume on the platform as

$$Z(s_{ld}^{i},t_{k})=\sum_{v\leq k}\sum_{s^{h}\in H(s^{u},s_{ld}^{i},t_{k})}x_{h}(s^{u},t_{v}),\;\forall s_{ld}^{i},k$$ (26)

It is known that the queuing length on the platform depends on the headway *τ*_*l*_, which means the larger the headway is, the longer the queuing length will be. The queuing length on the platform can be used to evaluate the service level of urban railway network. In the CTM, it is noted that the queuing length for each time interval is longest at the beginning of the current time interval, i.e., before flow transmission of each cell at each time interval, while it is shortest when at the ending of time interval, for the reason that some passengers queuing at the platform are transmitted at the ending of the current time interval.

To eliminate the above influence and obtain the reasonable queuing length to evaluate the service level, we define that the queuing length of each platform at time interval *t*_*k*_ means the total queuing flow during the time interval [*t*_*k*_Δ*T* − *τ*_*l*_,*t*_*k*_Δ*T*] with headway *τ*_*l*_, and it is calculated at the beginning of the above time interval. Thus, the queuing length includes the flows of the platform transmitted during [*t*_*k*_Δ*T* − *τ*_*l*_,*t*_*k*_Δ*T*] and the detained flow at the time interval *t*_*k*_. The length of time interval is Δ*T* and Δ*T* < *τ*_*l*_, so the headway *τ*_*l*_ may cover more than one time interval. It is known that the flow from Cell(*s*^*u*^) to $Cell(L_{ld},i,1),s_{ld}^{i}\in S(s^{u})$ at time interval *t*_*k*_ is that

$$M(s_{ld}^{i},t_{k})=\sum_{h=1}^{n}y_{h}(L_{ld},i,1,t_{k})-\sum_{s^{h}\in H(s_{ld}^{i+1},s_{ld}^{i+2},t_{k})}f_{h}(L_{ld},i,end,t_{k}),\;\forall s_{ld}^{i},k$$ (27)

The flow at the platform $s_{ld}^{i}$ transmitted during [*t*_*k*_Δ*T* − *τ*_*l*_,*t*_*k*_Δ*T*] is calculated as

$$\sum_{v=k-r+1}^{k}M(s_{ld}^{i},t_{v})+M(s_{ld}^{i},t_{k-r})\left(\tau_{l}-r\Delta T\right)/\Delta T$$ (28)

where *r* meets 0 ≤ *τ*_*l*_ − *r*Δ*T* < Δ*T*. As the detained flow on the platform in *t*_*k*_ is $Z(s_{ld}^{i},t_{k})$, so the queuing length on the platform in *t*_*k*_ is

$$Z(s_{ld}^{i},t_{k})+\sum_{v=k-r+1}^{k}M(s_{ld}^{i},t_{v})+M(s_{ld}^{i},t_{k-r})\left(\tau_{l}-r\Delta T\right)/\Delta T,\;\forall s_{ld}^{i},k$$ (29)

## Numerical examples

### Example 1: a network with three operating lines

#### Description

Take the urban rail network in **Fig 2** as an example. This network has three operating lines. Each line has four stations, and there are total nine stations in this urban rail network, including three transfer stations. The section mileage of each line is 1.6 km, the section travel times are all equal to 2.5 min, the minimum headways are all equal to 8 min, and the maximum passenger capacity of each train is 1000 passengers per train. The network operation period is from 6:00 to 23:00.

**Fig 4:**
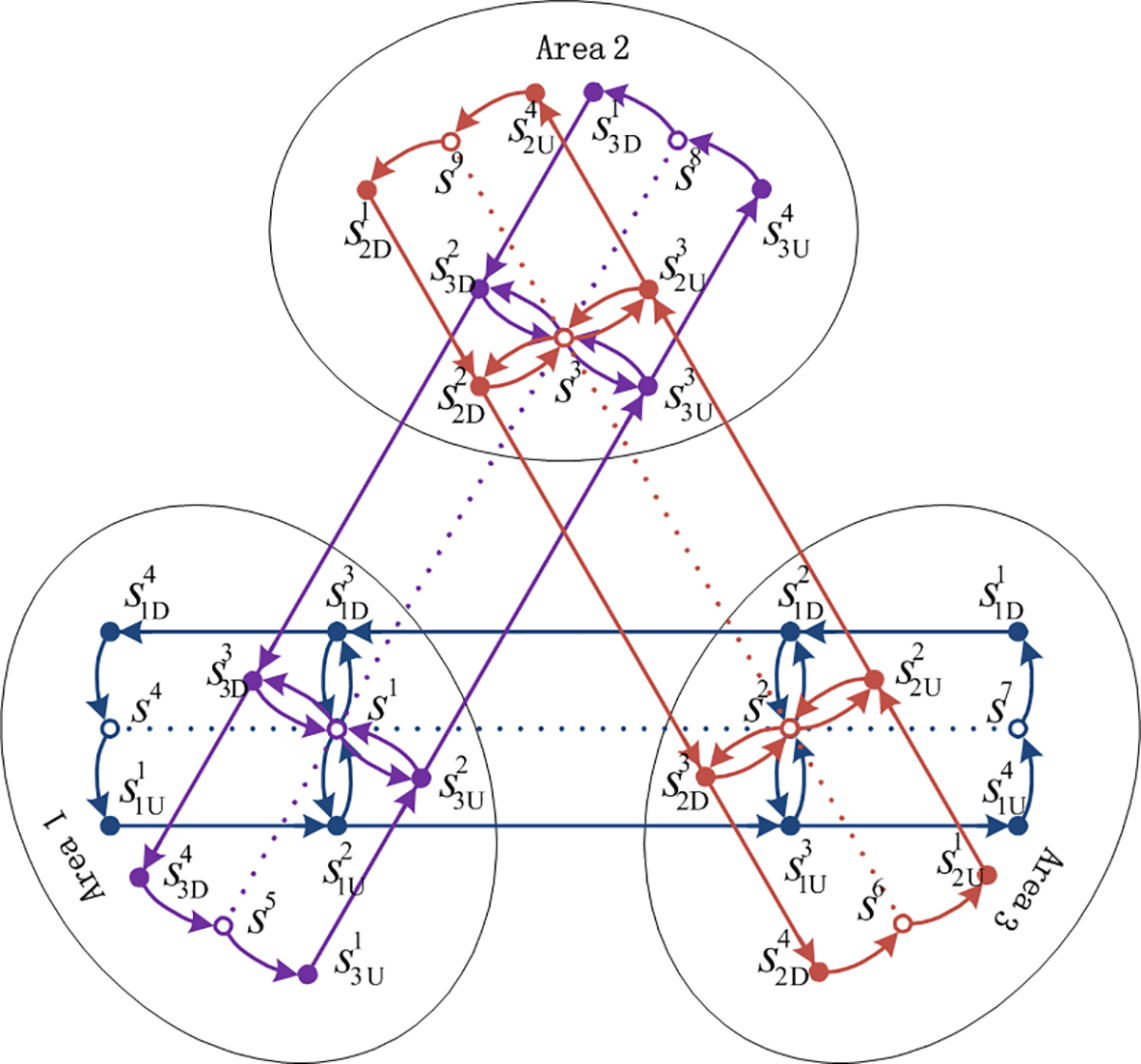
Three areas of the urban rail network.

**Fig 5:**
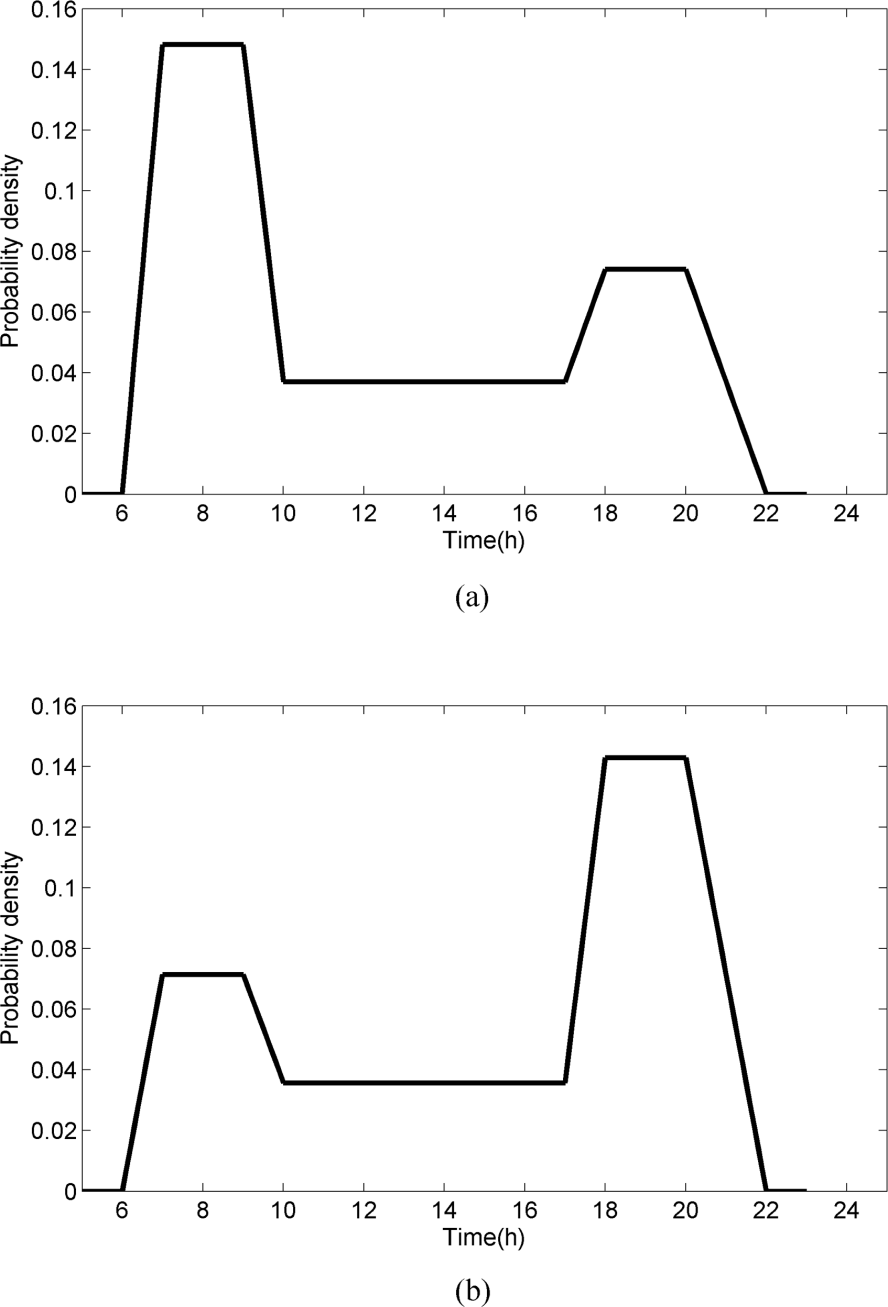
Two classes of density distributions of demands.

**Fig 4** shows that the network is divided into three areas, where area 1 is a Central Business District (CBD), while areas 2 and 3 are residential areas. The density distributions of O–D demands arriving in area 1 are shown in **Fig 5(A)**; the density distributions of O–D demands departing from area 1 are shown in **Fig 5(B)**. The demands within each area and between areas 2 and 3 are all equal to 0. The detailed O–D demands and density distributions are listed in **Table 1**. It can be seen from the density distributions of travel demands that all the morning peaks of O–D demands are the period from 7:00 to 9:00, while all the evening peaks of O–D demands are the period from 18:00 to 20:00. The O–D demands between areas 2 and 1 are larger than those between areas 3 and 1.

**Table 1:** O–D total demand and corresponding probability density distributions (unit: thousand persons).

O–D total demand		Area 1			Area 2			Area 3		
		1	4	5	2	6	7	3	8	9
Area 1	1				6.6(b)	5.5(b)	5.5(b)	5.5(b)	4.4(b)	4.4(b)
	4				5.5(b)	6.6(b)	6.6(b)	4.4(b)	5.5(b)	5.5(b)
	5				5.5(b)	6.6(b)	6.6(b)	4.4(b)	5.5(b)	5.5(b)
Area 2	2	6.6(a)	5.5(a)	5.5(a)						
	6	5.5(a)	6.6(a)	6.6(a)						
	7	5.5(a)	6.6(a)	6.6(a)						
Area 3	3	5.5(a)	4.4(a)	4.4(a)						
	8	4.4(a)	5.5(a)	5.5(a)						
	9	4.4(a)	5.5(a)	5.5(a)						

Set Δ*T* = 1.25 min and each section has two cells. Divide the operation period into 816 intervals by Δ*T*. The line transmission capacity in Δ*T* is Δ*T C*_*l*_/*τ*_*l*_ = 1.25 × 1000/8 = 156.25 passengers. Set parameters *λ* = 0.01, *θ* = 1, *η* = 0.8, *α* = 1, and the relative gap is 10^−3^.

With the computer (IntelCore 2.90GHz, 8GB RAM), we use Matlab (R2010b) to program and solve the model for this example, and it takes 19s CPU time to solve this model.

#### Analysis of indexes

The circulation volumes of all lines are listed in **Table 2**, and the circulation volume of the network is 7.92 10^5^ person kilometers.

**Table 2:** Operating line circulation volumes.

Line	Line 1	Line 2	Line 3
**Operating line circulation volume** **(person kilometer)**	3.40 × 10^5^	1.44 × 10^5^	3.08 × 10^5^

As some indexes are time-varying during an operation day, and the data are too large to be listed, we only calculate the statistical indexes about directional line $L_{1U}=\{s_{1U}^{1},s_{1U}^{2},s_{1U}^{3},s_{1U}^{4}\}$ to demonstrate and analyze the model.

**Fig 6:**
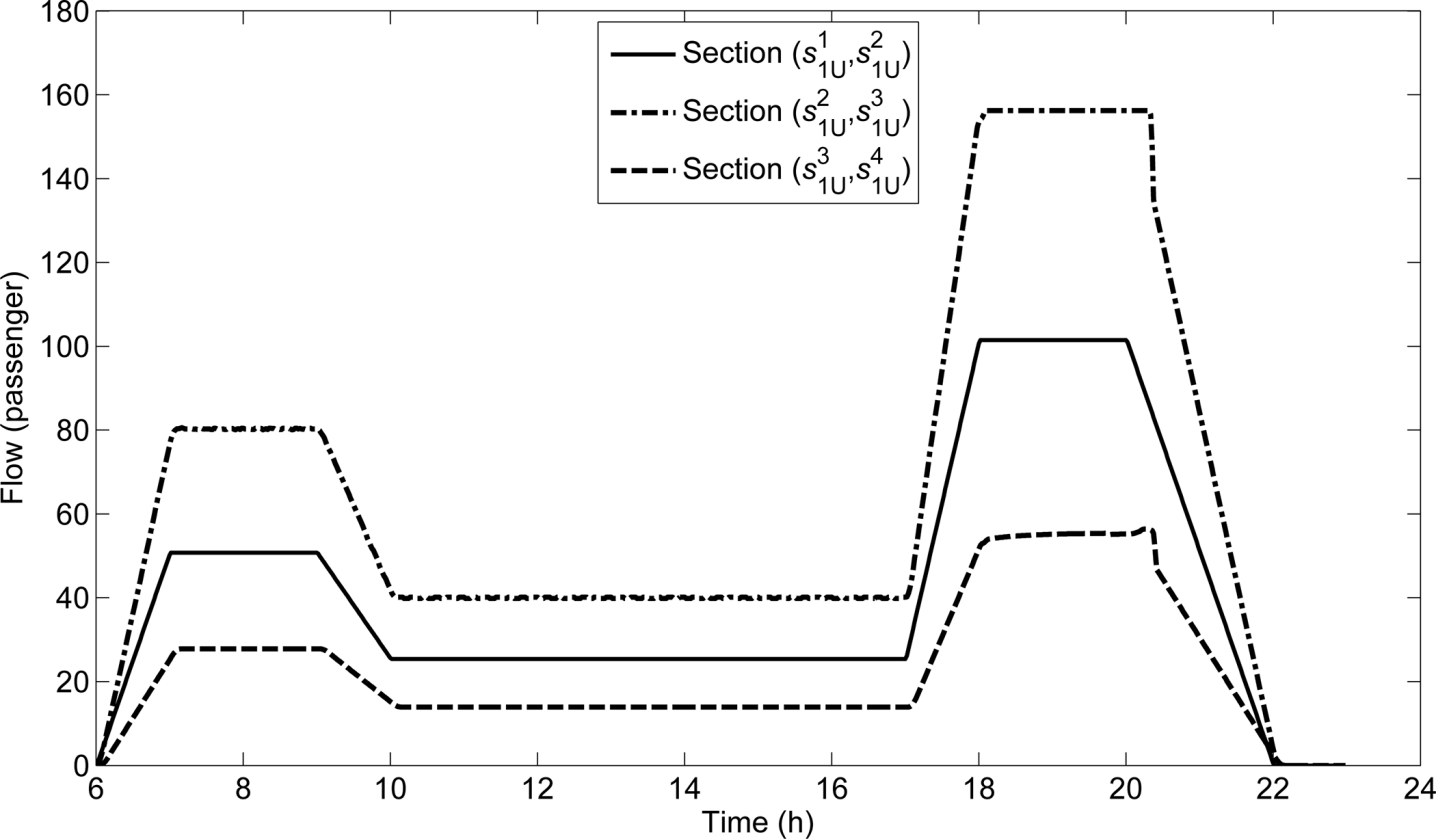
Section flows along *L*_1U_.

The time-varying section flow can be obtained by the method of MSA. There are three curves to show the time-varying section flows of $\left(s_{1U}^{1},s_{1U}^{2}),(s_{1U}^{2},s_{1U}^{3}),(s_{1U}^{3},s_{1U}^{4}\right)$ in **Fig 6**. Note that *L*_1U_ is a directional line in which passengers mainly depart from the CBD, and its travel demand distributions are shown in **Fig 5(B)**. The time-varying section flows are similar to **Fig 5(B)**. During evening peak hours, the section flow of $\left(s_{1U}^{2},s_{1U}^{3}\right)$ reaches transmission capacity 156.25 from 18:06:15 to 20:18:45. The section flow of $\left(s_{1U}^{3},s_{1U}^{4}\right)$ is the lowest among three curves, because there is no demand starting from ${\bar{s}}_{1U}^{3}$ along *L*_1U_.

**Fig 7:**
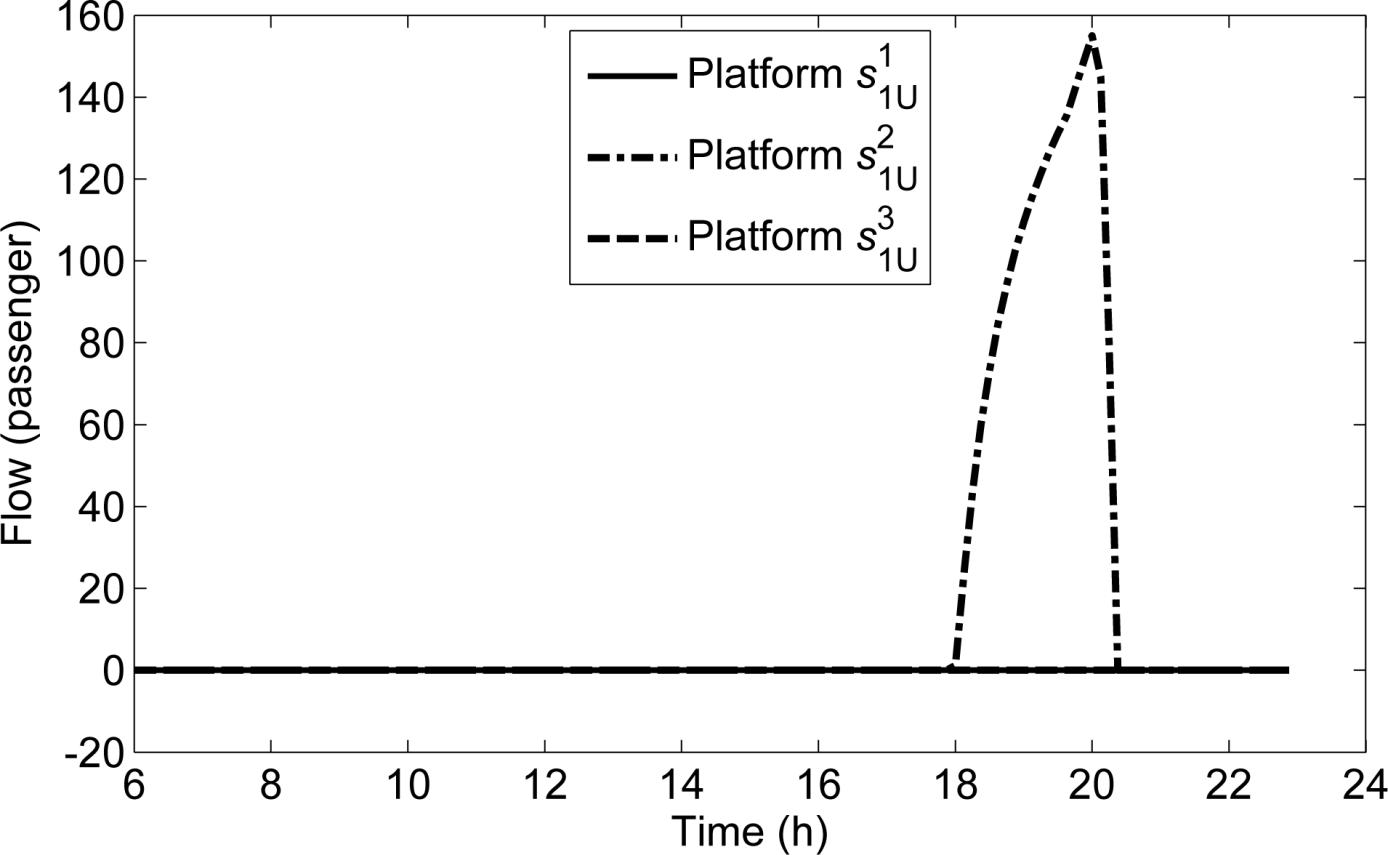
The detained flows of all platforms along *L*_1U_.

There are three curves to show the detained flows on $s_{1U}^{1},s_{1U}^{2},s_{1U}^{3}$ in **Fig 7**. The detained flow on $s_{1U}^{2}$ begins from 18:00:00, reaches the maximal value 155.91 at 20:02:30, and disappears at 20:20:00. It is for the reason that the demands from the upstream station ${\bar{s}}_{1U}^{1}=s^{4}$ occupies most capacity, which make the demands departing from ${\bar{s}}_{1U}^{2}$ during evening peak hours not be met.

**Fig 8:**
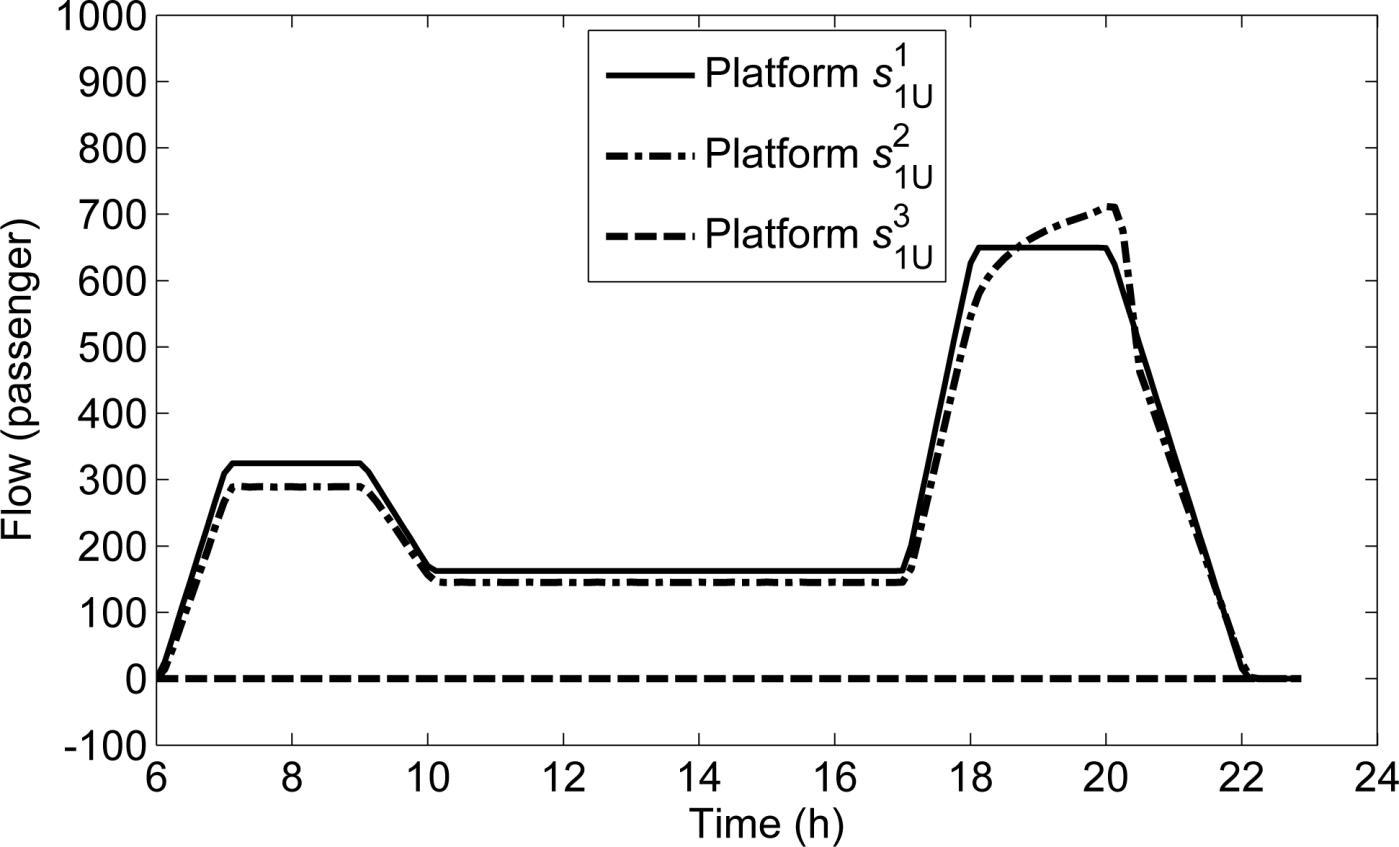
The queuing lengths of all platforms along *L*_1U_.

Three curves are illustrated in **Fig 8** to show the queuing lengths on $s_{1U}^{1},s_{1U}^{2},s_{1U}^{3}$. On $s_{1U}^{1}$, all passengers can be transmitted in time due to the sufficient capacity, then the queuing length distribution is similar to the demand distribution. On $s_{1U}^{2}$, the detained flow begins to appear from 18:00:00 due to the insufficient capacity. As the travel demand intensity will not change from 18:00:00, the queuing length continues to increasing. On $s_{1U}^{3}$, the queuing flow does not appear at any time bacause there is no departure and transfer passenger flow.

#### Analysis of passenger flow characteristics

To demonstrate the reasonability for dynamic assignment with the CTM, we show the path choice, the space priority principle and the flow moving among platforms of one station.

(a) Path choice

**Fig 9:**
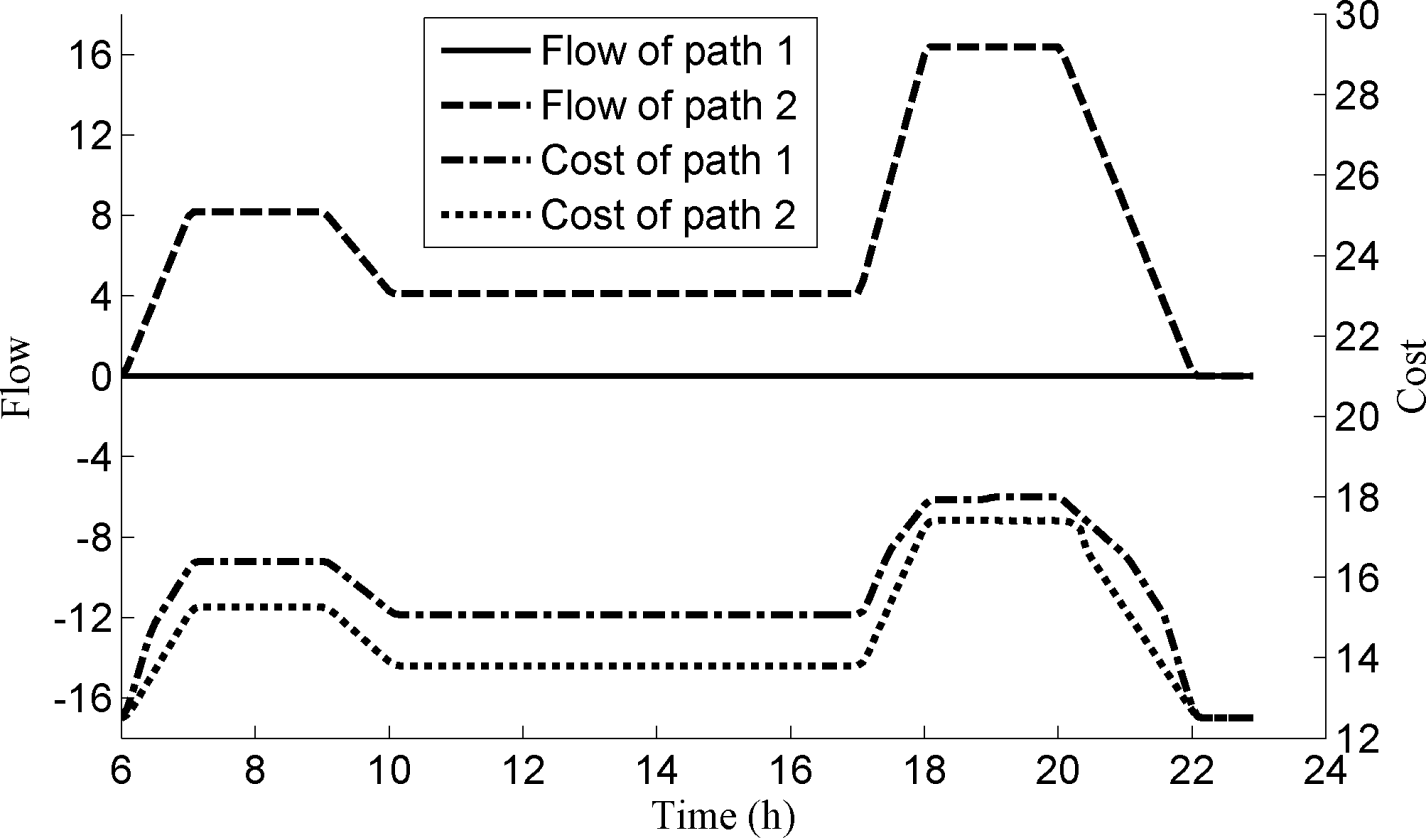
The flows and costs of two paths for passengers from platform $s_{1U}^{2}$ to station *s*^9^.

As passengers follows the instantaneous dynamic route choice principle, we now analyze the path choice for the passengers from platform $s_{1U}^{2}$ to station *s*^9^. We adopt two paths, i.e., path 1: $\left(s_{1U}^{2},s^{1},s_{3U}^{2},s_{3U}^{3},s^{3},s_{2U}^{3},s_{2U}^{4},s^{9}\right)$ and path 2: $\left(s_{1U}^{2},s_{1U}^{3},s^{2},s_{2U}^{2},s_{2U}^{3},s_{2U}^{4},s^{9}\right)$. The flows and costs of these two paths are shown in **Fig 9**, we can see that the costs of path 1 for all time intervals are larger than those of path 2, so all passengers choose the path 2 to travel to *s*^9^.

**Fig 10:**
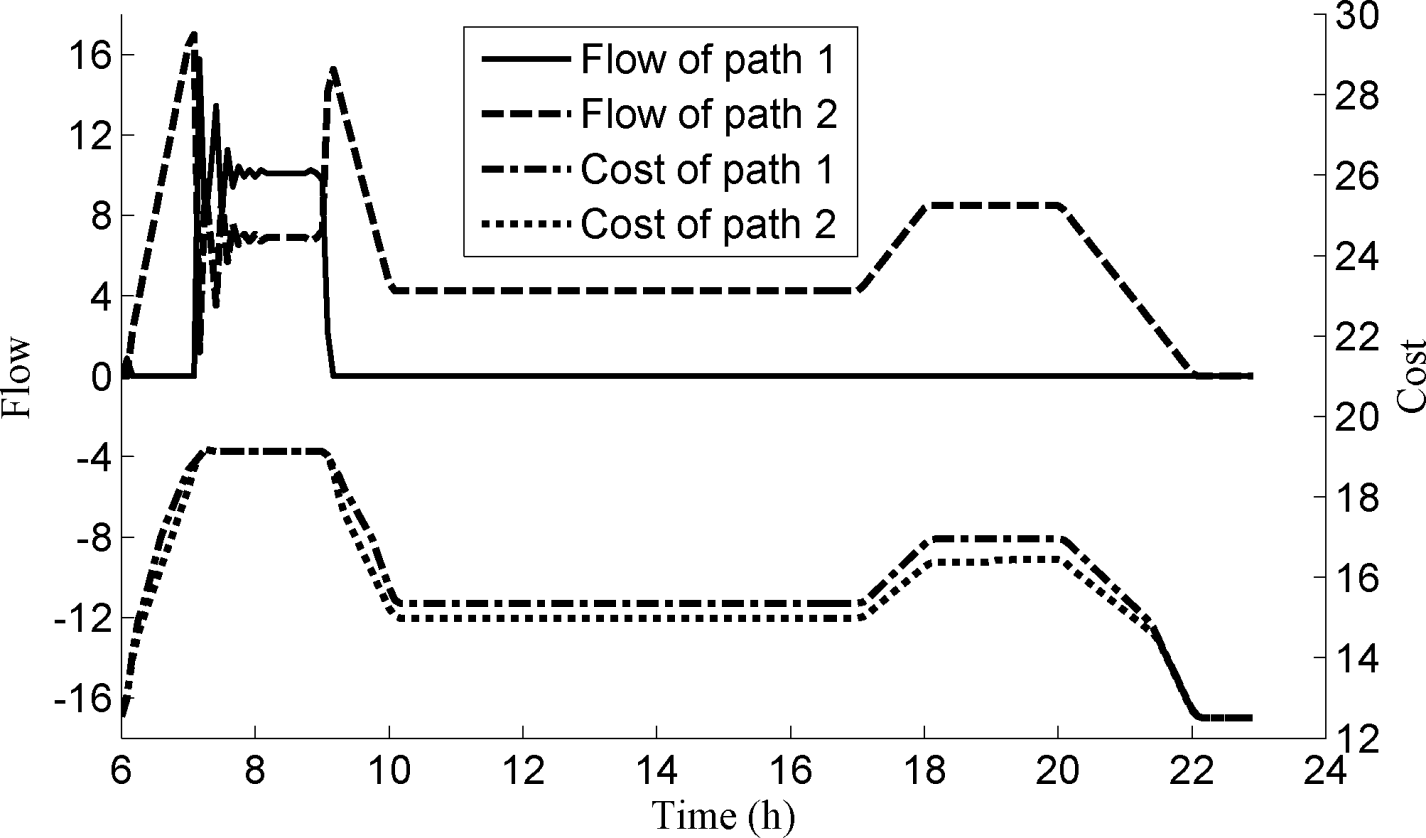
The flows and costs of two paths for passengers from platform $s_{2D}^{2}$ to station *s*^4^.

Now we consider the path choice for the passengers from platform $s_{2D}^{2}$ to station *s*^4^. We also adopt two paths, i.e., path 1: $\left(s_{2D}^{2},s^{3},s_{3D}^{2},s_{3D}^{3},s^{1},s_{1D}^{3},s_{1D}^{4},s^{4}\right)$ and path 2: $\left(s_{2D}^{2},s_{2D}^{3},s^{2},s_{1D}^{2},s_{1D}^{3},s_{1D}^{4},s^{4}\right)$. **Fig 10** shows the costs and the flows of these two paths. We can see that the costs of the two paths are equal during the time period [07:08:45, 09:06:15], and the flows of the two paths are larger than zero. In other time periods, the costs of path 1 are larger than those of path 2, so the passengers all choose the path 2.

From **Figs 9 and 10**, it is obvious that the dynamic flow of the network is in an instantaneous dynamic user equilibrium state.

(b) The space priority principle

**Fig 11:**
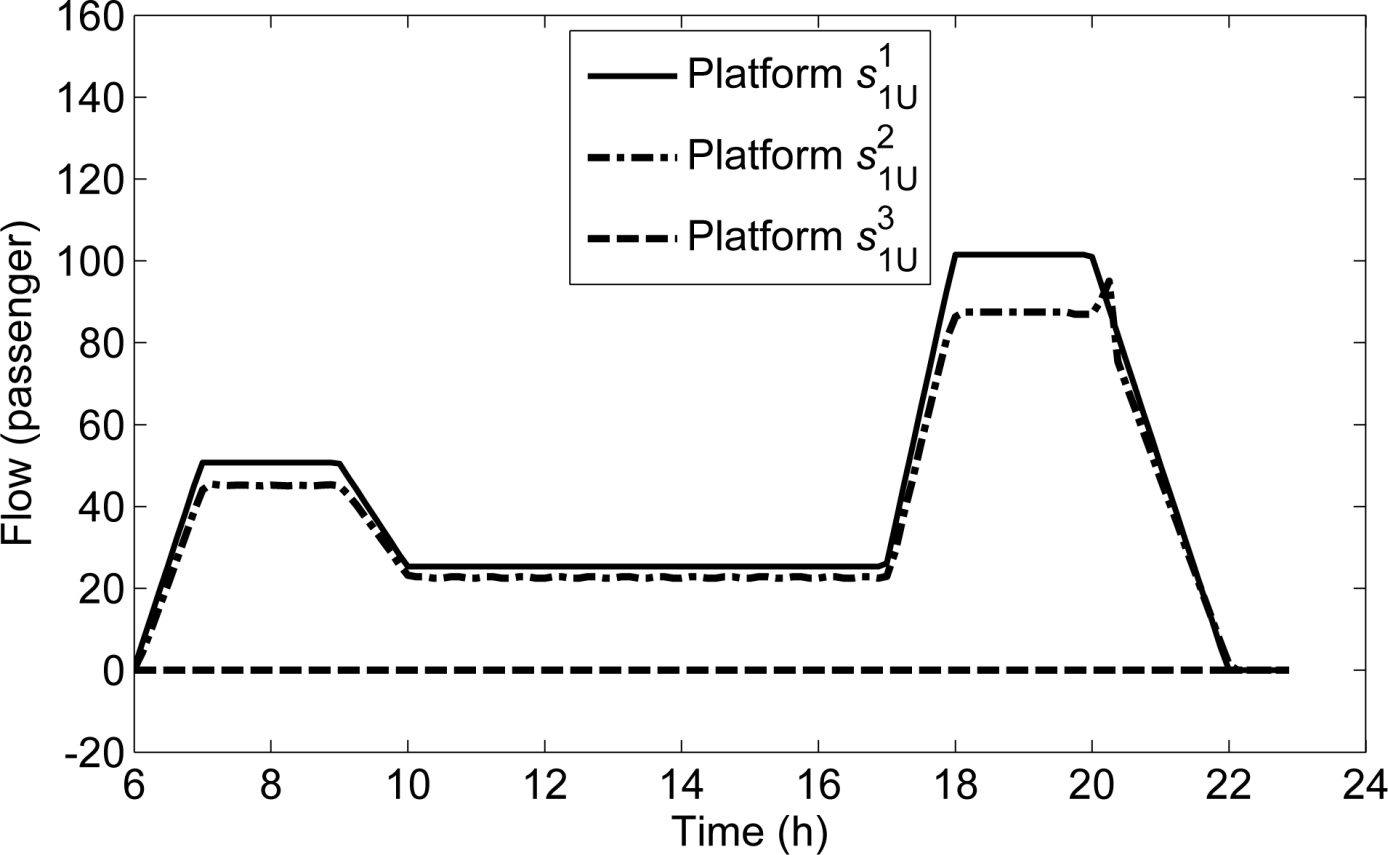
The transmitted flows of all platforms along *L*_1U_.

**Fig 11** shows the curves of the transmitted passenger flows on $s_{1U}^{1},s_{1U}^{2},s_{1U}^{3}$ along directional line *L*_1U_. The flow on $s_{1U}^{3}$ is 0, and the flow on $s_{1U}^{1}$ shows that the transmitted passenger flow is consistent with the departure demand from *s*^1^. The transmitted flow on $s_{1U}^{2}$ increases sharply from 20:02:30.

**Fig 12:**
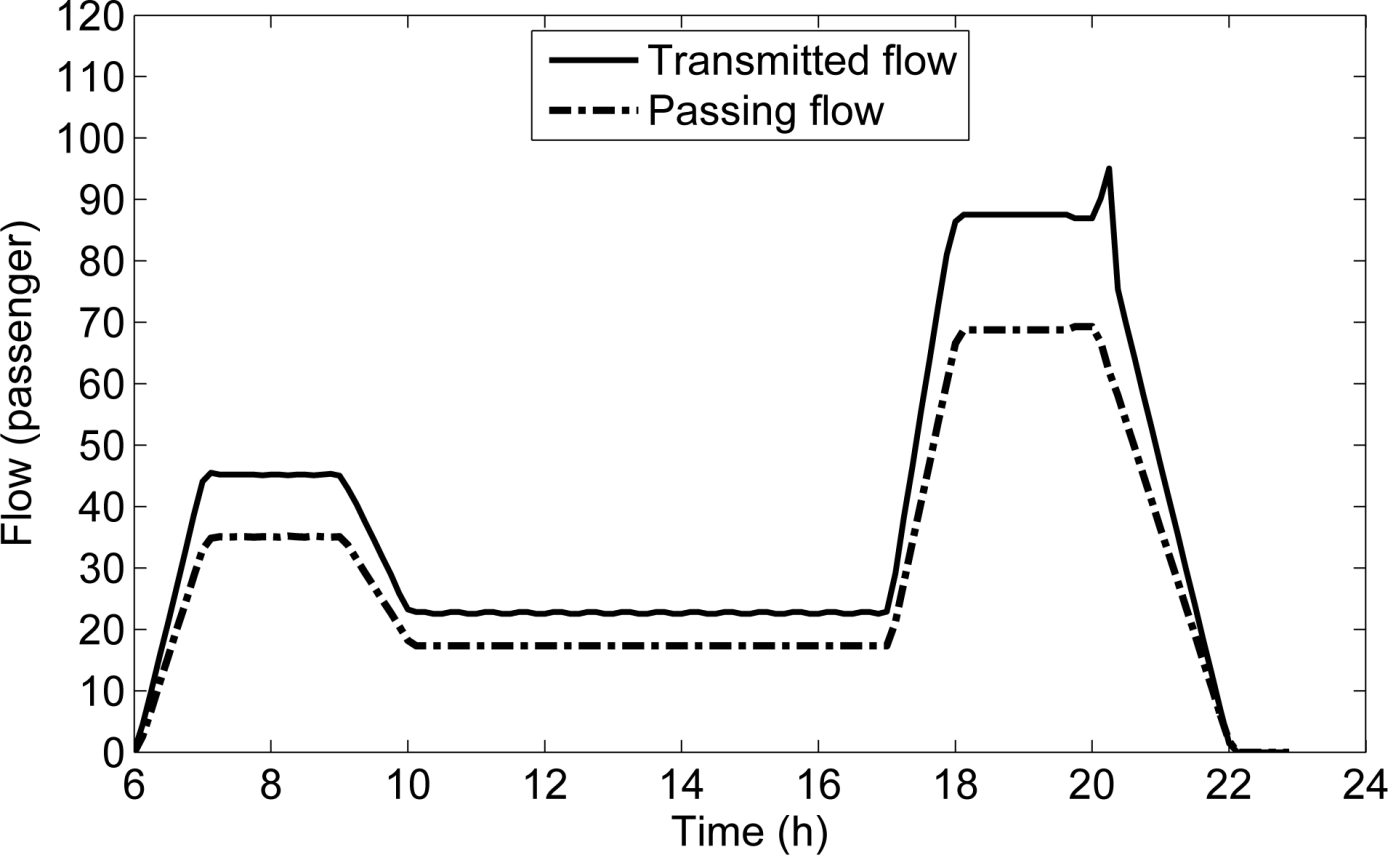
Transmitted and passing flows on $s_{1U}^{2}$ along *L*_1U_.

To explain this phenomenon, we compare the curve of the transmitted flow on $s_{1U}^{2}$ with that of the passing flow in **Fig 12**. As shown in **Fig 12**, the passing flow on $s_{1U}^{2}$ decreases from 20:02:30, while the transmitted flow on $s_{1U}^{2}$ increases. Moreover, it can be seen in **Fig 7** that there is no detained flow at $s_{1U}^{1}$ at any time, while the detained flow on $s_{1U}^{2}$ begins to appear from 18:00:00 to 20:20:00. With the space priority principle, when the capacity for $s_{1U}^{1}$ is sufficient, the flow on $s_{1U}^{1}$ can all be transmitted; when the capacity for $s_{1U}^{2}$ is insufficient, the flow on $s_{1U}^{2}$ can be transmitted according to the remaining capacity, then there are some detained flow on $s_{1U}^{2}$. It can be seen in **Fig 12** that the sum of the transmitted flows and the passing flows of $s_{1U}^{2}$ at each time interval from 18:00:00 to 20:20:00, are exactly equal to the line transmission capacity (156.25 passengers per the unit time Δ*T*). Thus, the reason for the phenomenon is that the decrease of demand in the upstream platform $s_{1U}^{1}$ makes the passing volume of $s_{1U}^{2}$ decline, and the increased remaining capacity can be used to transmit the detained passengers on $s_{1U}^{2}$, which makes the transmitted flow on $s_{1U}^{2}$ increase sharply. This exactly reflects the space priority principle.

(b) Flow moving among platforms of the station

**Fig 13:**
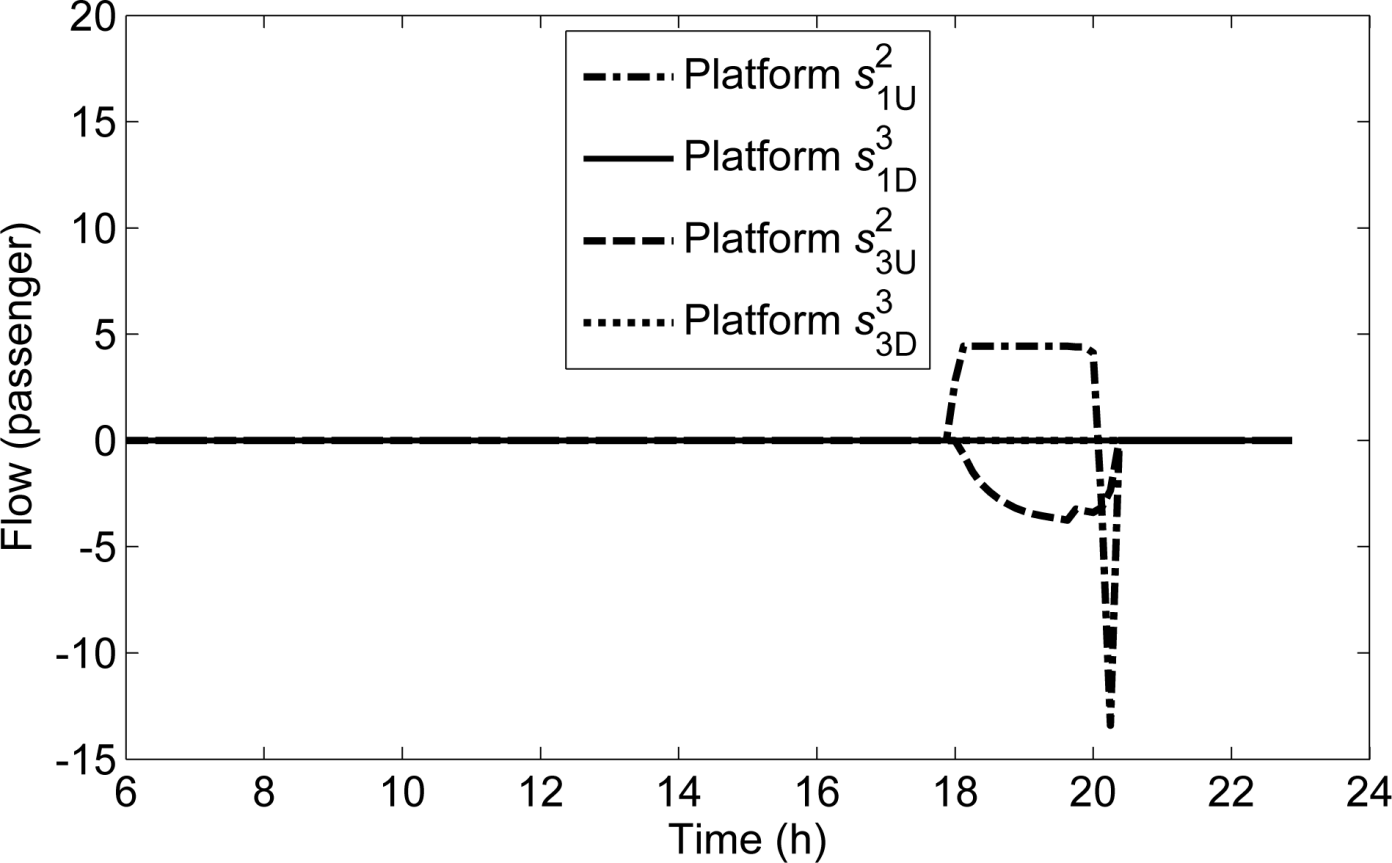
Curves of differences between the non-detained and transmitted flows of all platforms in *s*^1^.

In each time interval, there are differences between the non-detained flow and the transmitted flow on the platform. The non-detained flow includes O-D demand departing from the platform and the transfer flow arriving at the platform in the current time interval, and it does not include the detained passenger flow. If the non-detained flow is equal to the transmitted flows, the detained flow in the current time interval is equal to that at the previous time interval. If the non-detained flow is larger than the transmitted flow, the detained flow at the current time interval is larger than that at the previous time interval. If the non-detained demand is less than the transmitted flow, the detained flow at the current time interval is less than that at the previous time interval. The curves of the differences between the non-detained flow and the transmitted flow on four platforms in *s*^1^ are illustrated in **Fig 13**.

On $s_{1D}^{3},s_{3D}^{3}\in S(s^{1})$, the value are 0, which indicates that the non-detained flows at any time are not restricted by the capacity. On $s_{1U}^{2}\in S(s^{1})$, the value in the period from 17:58:45 to 20:03:45 is larger than 0, which indicates that the capacity in this period cannot satisfy the non-detained flow, and the detained flow appears. The value in the period from 20:05:00 to 20:20:00 is less than 0, which indicates that the capacity in this period exceeds the non-detained demand, and the detained flow is transmitted. Note that the values of $s_{1U}^{2}$ before 17:58:45 and after 20:20:00 are both equal to 0, i.e., the detained flows in the two periods are both equal to 0.

In view of the figure area, the detained flow from 17:58:45 to 20:03:45 is much larger than the transmitted flow from 20:05:00 to 20:20:00 on the platform $s_{1U}^{2}$, but the detained flow disappear after 20:20:00. To explain this phenomenon, we can see that for $s_{3U}^{2}\in S(s^{1})$, the values in the period from 18:00:00 to 20:20:00 are less than 0, that is, the non-detained flow in this period is less than the transmitted flows. At other times, the non-detained demand is equal to the transmitted flows. It is obvious that some detained passengers on $s_{1U}^{2}$ change their travel route, and move to $s_{3U}^{2}$. In view of the figure area, the sum of the two negative areas is equal to the positive area.

#### Sensitivity analysis

**Fig 14:**
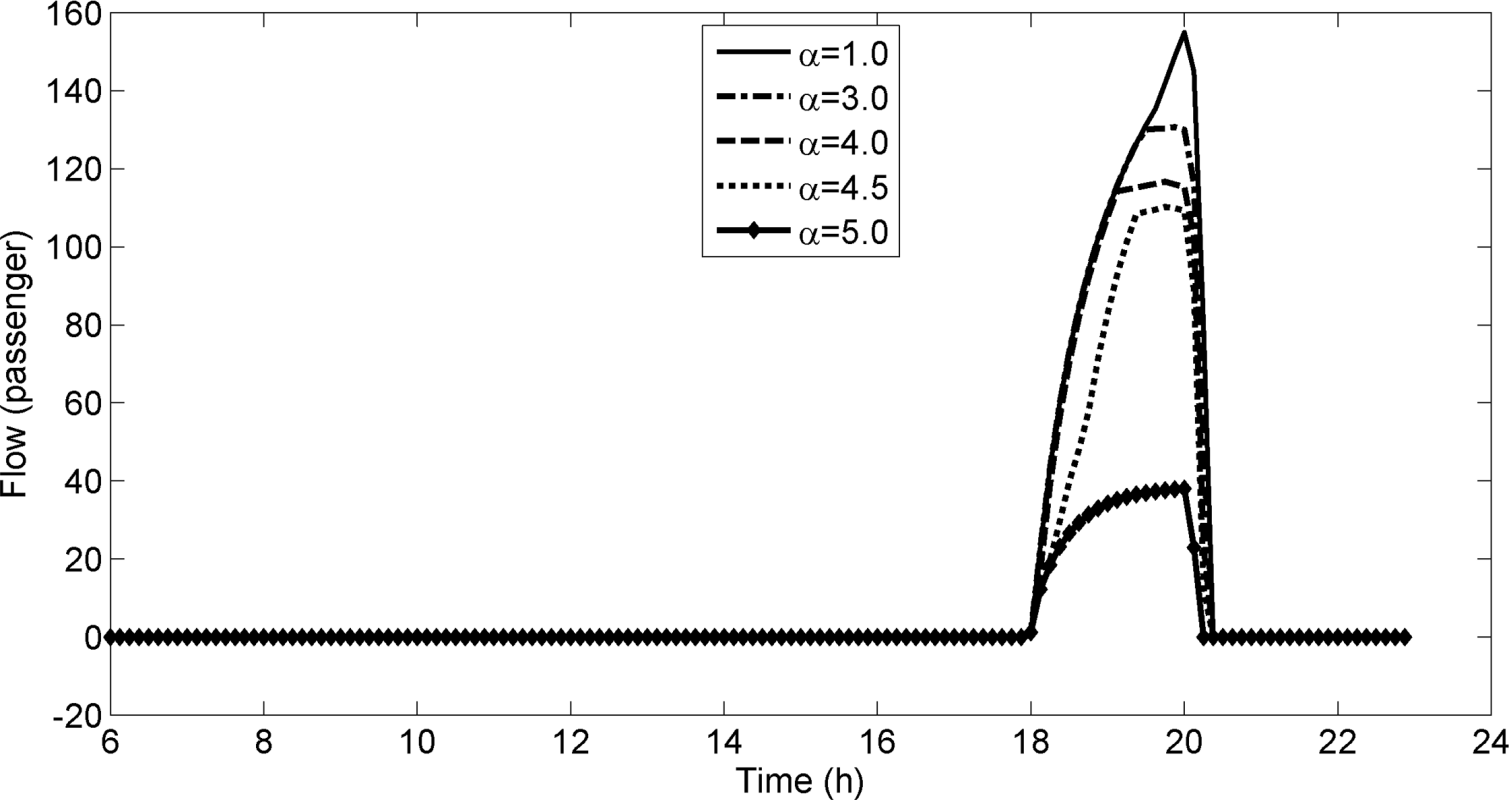
The detained flows of platform $s_{1U}^{2}$ for different values of parameter *α*.

We set the parameter *α* = 1.0,3.0,4.0,4.5,5.0 respectively, and don’t change other parameters. The detained flows of platform $s_{1U}^{2}$ are calculated by the CTM for different values of parameter *α*, shown in **Fig 14**. From **Fig 14**, It can be seen that the detained flows of platform $s_{1U}^{2}$ decrease with the increasing of the parameter *α*. From the demand distributions in Table 1, we know that the demands from Area 1 to Area 2 are larger than those from Area 1 to Area 3. It results in that the congestion in *L*_1U_ is more than that in *L*_3U_. With the increase of parameter *α*, the cost differences between the paths traveling from Area 1 to Area 2 and from Area 1 to Area 3 increase. It results in that the flows change their paths and the flows shift from paths traveling from Area 1 to Area 2 to those traveling from Area 1 to Area 3. Thus, the increase of parameter *α* makes the decrease of the detained flows of platform $s_{1U}^{2}$.

**Fig 15:**
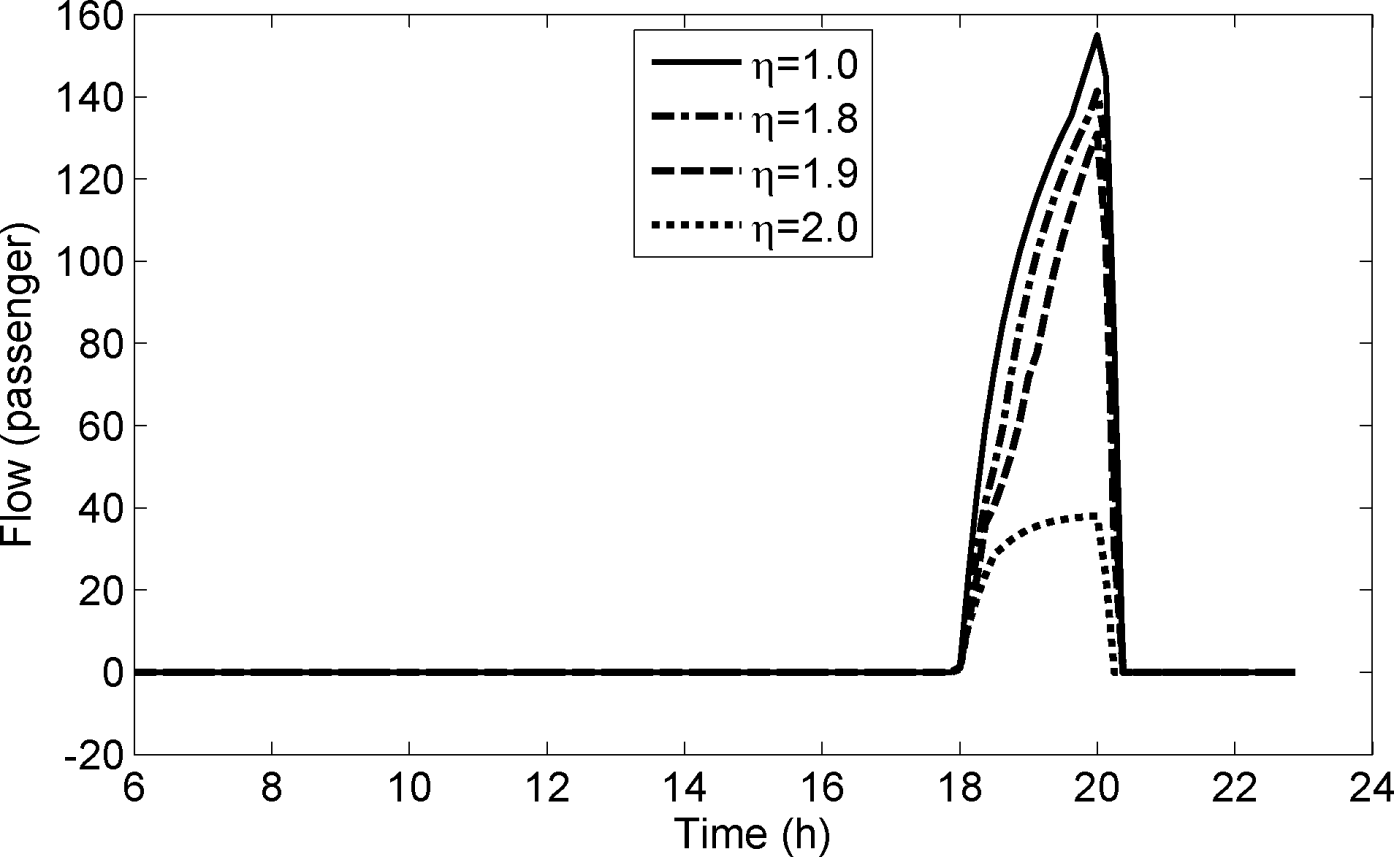
The detained flows of platform $s_{1U}^{2}$ for different values of parameter *η*.

For parameter *η*, we now do the sensitivity analysis. Set parameter *η* = 1.0,1.8,1.9,2.0 and don’t change other parameters. We also calculate the detained flows of platform $s_{1U}^{2}$ for different values of parameter *η*. **Fig 15** shows the curves of the detained flows of platform $s_{1U}^{2}$ and we can see that the detained flow of platform $s_{1U}^{2}$ decreases with the increasing of the parameter *α*. The reason is similar to that of parameter *α*. It results in that the cost differences between the paths traveling from Area 1 to Area 2 and from Area 1 to Area 3 increase with the increase of parameter *η*. Thus, the increase of parameter *η* makes the decrease of detained flows of platform $s_{1U}^{2}$.

### Example 2: Beijing Metro network

We take the Beijing Metro network as an example to illustrate the CTM. The total mileage of the network is 414.503 km, the number of stations in the network is 231, including 40 transfer stations, and the network has 15 lines, i.e. Line 1, Line 2, Line 4, Line 5, Line 6, Line 8, Line 9, Line 10, Line 13, Line 14, Line 15, Batong Line, Fangshan Line, Changping Line and Yizhuang Line. Line 2 and Line 10 are annular lines, while the other 13 lines are linear. The minimum headways are all 2.5 min, and the maximum passenger capacity of each train is 1200 passengers per train. The network operation period is from 6:00 to 23:00. The total amount of all O–D demands is 5.496 × 10^6^.

We set Δ*T* = 1 min, and the total number of time intervals is 1020. The transmission capacity of a line within Δ*T* is that $\Delta T\;C_{l}^{max}/\tau_{l}=1\times \frac{1200}{2.5}=480$. The relative gap is 10^−3^, and the total CPU time is 5.16h.

The circulation volumes of all lines are illustrated in **Table 3**. The total circulation volume of the network is 8.69 × 10^7^ person kilometers.

**Table 3:** Operating line circulation volumes of all lines in Beijing Metro network.

**Line**	Line 1	Line 2	Line 4	Line 5	Line 6
**Operating line circulation volume** **(person kilometer)**	1.08s10^7^	6.06 × 10^6^	7.71 × 10^6^	6.70 × 10^6^	4.93 × 10^6^
**Line**	Line 8	Line 9	Line 10	Line 13	Line 14
**Operating line circulation volume** **(person kilometer)**	2.40s10^6^	2.42 × 10^6^	2.40 × 10^7^	9.92 × 10^6^	2.97 × 10^5^
**Line**	Line 15	Batong Line	Changping Line	Fangshan Line	Yizhuang Line
**Operating line circulation volume** **(person kilometer)**	2.41s10^6^	3.74 × 10^6^	1.91 × 10^6^	1.54 × 10^6^	2.15 × 10^6^

**Fig 16:**
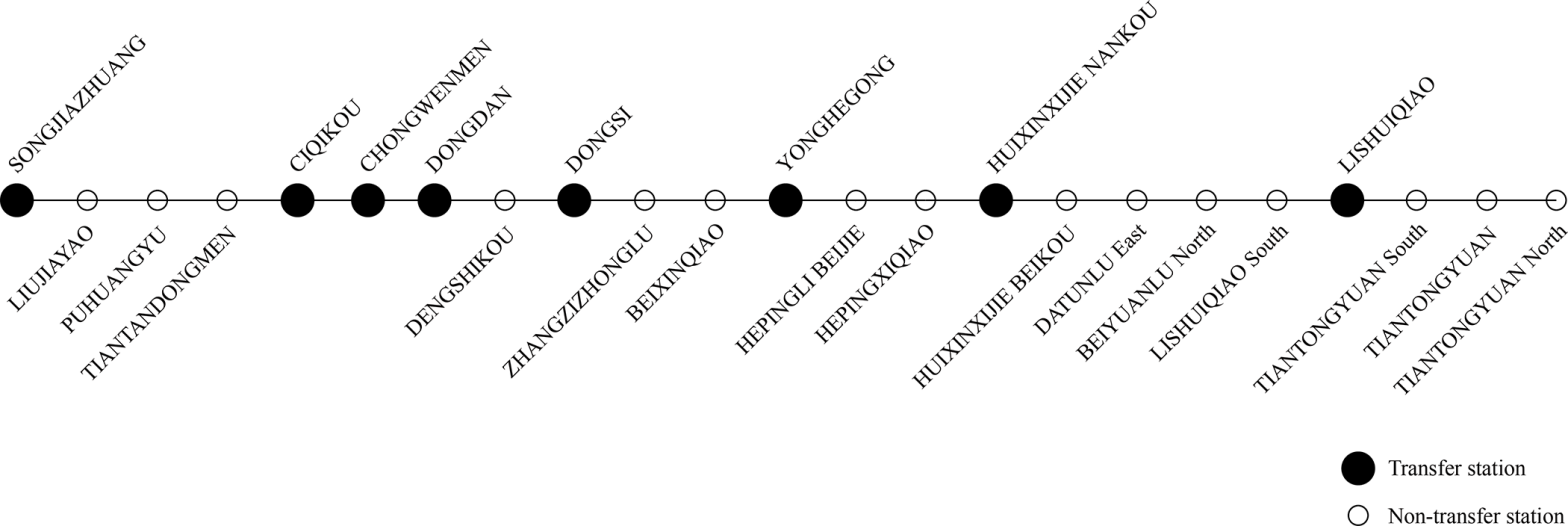
Illustration of Line 5.

We take Line 5 as an example to analyze the space-time flow distribution. There are 23 stations in Line 5 illustrated in **Fig 16**, where solid dots represent transfer stations, while hollow dots represent the non-transfer stations. There are 7 transfer stations including Songjiazhuang, Ciqikou, Chongwenmen, Dongdan, Dongsi, Yonghegong and Huixinxijie Nankou in Line 5.

**Fig 17:**
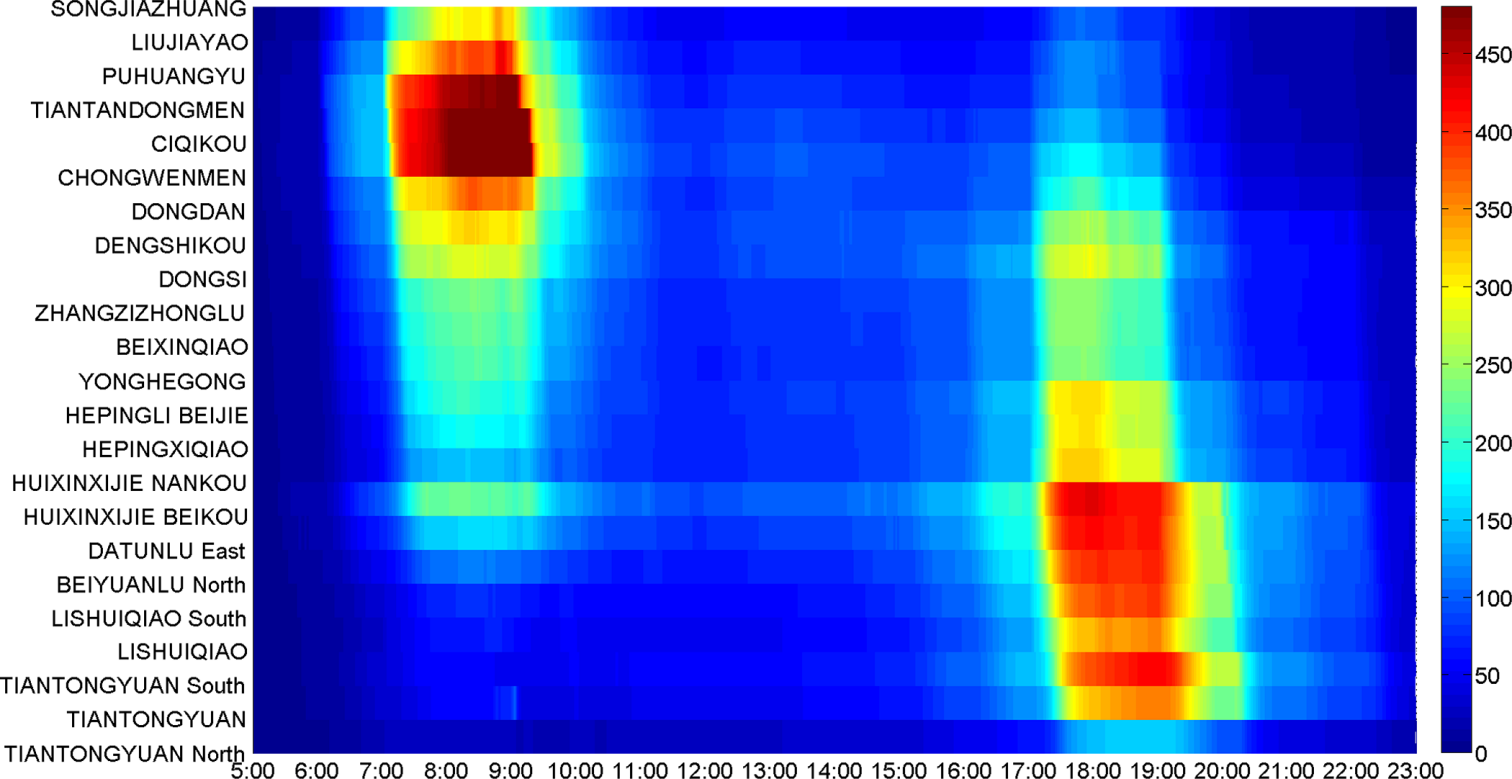
The space-time section flow distribution from Songjiazhuang to Tiantongyuan North.

**Fig 18:**
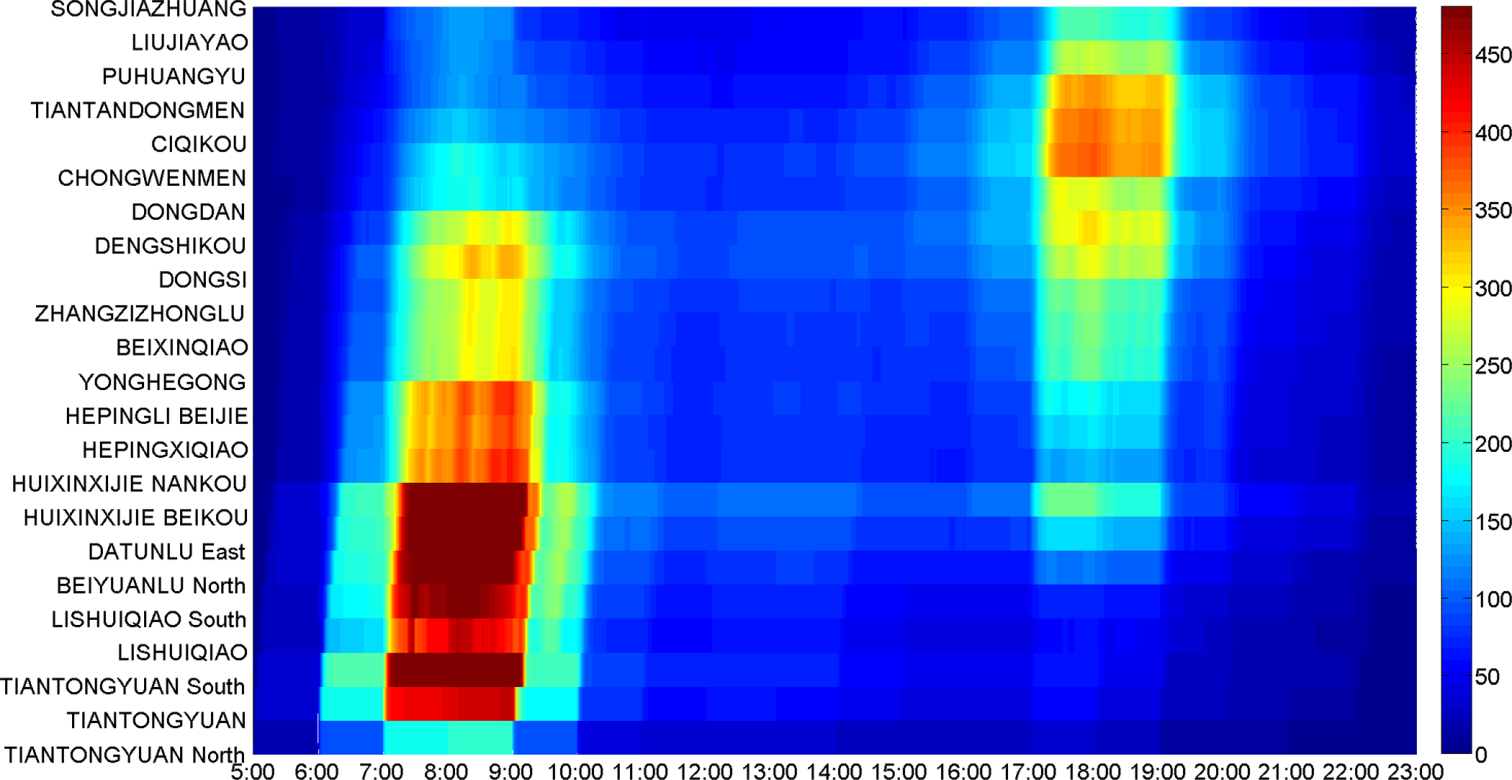
The space-time section flow distribution from Tiantongyuan North to Songjiazhuang.

**Fig 17** illustrates the space-time section flow distribution in the direction from Songjiazhuang to Tiantongyuan North, while **Fig 18** illustrates that in the opposite direction. Comparing **Fig 17** with **Fig 18**, there are a morning peak in the sections from Songjiazhuang to Dongsi in **Fig 17**, and an evening peak in the sections from Dongsi to Songjiazhuang in **Fig 18**. There are an evening peak in the sections from Dongdan to Huixinxijie Nankou in **Fig 17**, and a morning peak in the sections from Huixinxijie Nankou to Dongdan in **Fig 18**. The space-time flow distributions in the two directions have the character of symmetry in travel time, and show the tidal traffic flow phenomenon. The passenger flow of the morning peak from Songjiazhuang to Dongsi are more intensive than those of the evening peak from Dongsi to Songjiazhuang. As the morning peak is the time period when passengers need to get to their workplaces at specified times, while the evening peak is the time period of going off duty, so the travel time period chosen by the passengers is relatively wider. As shown in **Fig 17** and **Fig 18**, the section flow at transfer stations will vary greatly, because the section flow can gather from other lines, or disperse to other lines via the transfer stations. In addition, the density of the passenger flow space-time distribution in each section is less than 480, so it satisfies the capacity constraints.

**Fig 19:**
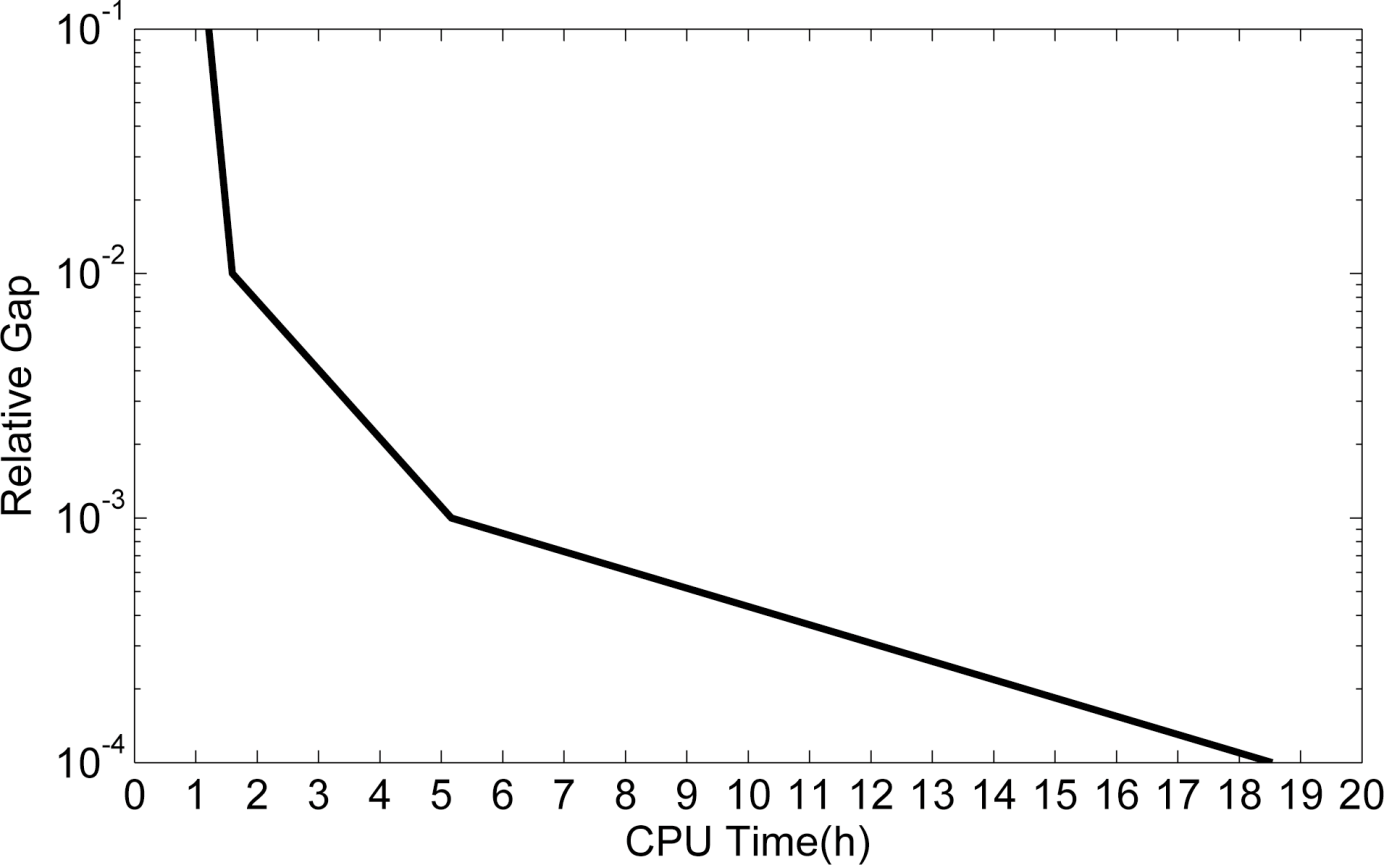
The variation of CPU time with relative gap.

**Fig 19** shows the variation of CPU time with the relative gap. We can see that the convergence speed is fast with a low relative gap, then as the relative gap gets lower, the CPU time becomes longer, and the convergence speed gets slower. When the relative gap reaches 10^−4^, the CPU time reaches 18.5h, for the reason that the algorithm of MSA takes a long time to reach a high relative gap.

## Conclusions

In this paper, the concept of continuous transmission is introduced to model the dynamic assignment for the urban rail network without the restriction of train schedules. Based on the cell transmission mechanism, the proposed CTM considers the priority principle, queuing process, capacity constraints and congestion effect. Using the MSA, the instantaneous dynamic optimal state can be reached at each interval. A fast and effective method is designed for solving the shortest path for the urban rail network. This method decreases the computing cost for solving the CTM, and it is applied efficiently to the large-scale urban rail network.

The CTM can generate some important evaluation indexes, including the time-varying section flow, the circulation volume, the detained flow on the platform, and the queuing length on the platform. It provides effective supports for optimizing the space-time resource allocation for the urban rail network. Finally, the model and its potential application are demonstrated via two numerical experiments using a small-scale network and the Beijing Metro network.

The proposed method is assumed that the passengers follow the instantaneous dynamic route choice principle, and as a topic of further interest, we can explore the CTM based on the continuous transmission with predictive/ideal dynamic user equilibrium. In the future studies, this method of DAURTN can be employed for optimizing the schedule, frequency and rolling stock, and evaluating the transportation capacity of network.

## Supporting information

S1 Table No. of each station. (XLSX)

S2 Table The distance between two adjacent stations. (XLSX)

S3 Table No. of each line. (XLSX)

S4 Table Line style. (XLSX)

S5 Table Total distance and average running speed of each line. (XLSX)

S6 Table The stations of each line passing. (XLSX)
